# Agrobacterium tumefaciens Deploys a Versatile Antibacterial Strategy To Increase Its Competitiveness

**DOI:** 10.1128/JB.00490-20

**Published:** 2021-01-11

**Authors:** Manda Yu, Yi-Chieh Wang, Ching-Jou Huang, Lay-Sun Ma, Erh-Min Lai

**Affiliations:** a Institute of Plant and Microbial Biology, Academia Sinica, Taipei, Taiwan; Brigham and Women's Hospital/Harvard Medical School

**Keywords:** type VI secretion system, effector, peptidoglycan amidase, interbacterial competition, *Agrobacterium tumefaciens*

## Abstract

The type VI secretion system (T6SS) is a widespread antibacterial weapon capable of secreting multiple effectors for inhibition of competitor cells. Most of the effectors in the system share the same purpose of target intoxication, but the rationale for maintaining various types of effectors in a species is not well studied. In this study, we showed that a peptidoglycan amidase effector in Agrobacterium tumefaciens, Tae, cleaves d-Ala-*meso*-diaminopimelic acid (mDAP) and d-Glu bonds in peptidoglycan and is able to suppress the growth of Escherichia coli recipient cells. The growth suppression was effective only under the condition in which E. coli cells are actively growing. In contrast, the Tde DNase effectors in the strain possessed a dominant killing effect under carbon starvation. Microscopic analysis showed that Tde triggers cell elongation and DNA degradation, while Tae causes cell enlargement without DNA damage in E. coli recipient cells. In a rich medium, A. tumefaciens harboring only functional Tae was able to maintain competitiveness among E. coli and its own sibling cells. Growth suppression and the competitive advantage of A. tumefaciens were abrogated when recipient cells produced the Tae-specific immunity protein Tai. Given that Tae is highly conserved among A. tumefaciens strains, the combination of Tae and Tde effectors could allow A. tumefaciens to better compete with various competitors by increasing its survival during changing environmental conditions.

**IMPORTANCE** The T6SS encodes multiple effectors with diverse functions, but little is known about the biological significance of harboring such a repertoire of effectors. We reported that the T6SS antibacterial activity of the plant pathogen Agrobacterium tumefaciens can be enhanced under carbon starvation or when recipient cell wall peptidoglycan is disturbed. This led to a newly discovered role for the T6SS peptidoglycan amidase Tae effector in providing a growth advantage dependent on the growth status of the target cell. This is in contrast to the Tde DNase effectors that are dominant during carbon starvation. Our study suggests that combining Tae and other effectors could allow A. tumefaciens to increase its competitiveness among changing environmental conditions.

## Abstract (precis)

The T6SS encodes multiple effectors with diverse functions, but little is known about the biological significance of harboring such a repertoire of effectors. We reported that the T6SS antibacterial activity of the plant pathogen Agrobacterium tumefaciens can be enhanced under carbon starvation or when recipient cell wall peptidoglycan is disturbed.

## INTRODUCTION

The type VI secretion system (T6SS) is a contractile secretion apparatus used by many Gram-negative bacteria to deliver effectors into target cells or extracellular milieu for the benefits of virulence, interbacterial competition, or metal ion acquisition (1). T6SS in different bacterial species is regulated by various types of environmental signals (2). Various T6SS effectors in many characterized species are secreted at the same time or differentially regulated (3). One example is the Pseudomonas aeruginosa effector Tse4, which is most active in high-salinity environments and synergizes with other effectors to maximize antibacterial activity (4). Thus, delivery of a cocktail of effectors can serve as a bet-hedging strategy in variable environmental conditions. Some effectors display a target-specific property to eliminate certain target cell types that respond only to a specific incoming effector. The T6SS effector Ssp2 from Serratia marcescens strain Db10 requires the presence of the recipient target cell protein DsbA for its toxic action (5). This indicates that the external environmental conditions, as well as the target cell genotypes, play critical roles for specific effectors to dominantly act against favorable targets. However, how T6SS-possessing bacteria coordinate the function of different effectors in response to different environmental cues to secure their competitive growth advantages remains unclear. In this study, a plant pathogen Agrobacterium tumefaciens, which deploys the type IV secretion system (T4SS) for pathogenesis and T6SS for interbacterial competition (6–8), was used to tackle this question.

A. tumefaciens is a plant pathogen and an important tool in genetic modification of plants owing to its ability to transfer its own DNA and integrate into the plant genome through T4SS (6). Besides T4SS, T6SS is also widespread in A. tumefaciens species with a conserved function for interbacterial competition (6–9). A. tumefaciens strain C58 has been used as a model for studying T6SS because of its completed genome and well-established genetic tools and resources (10). It contains one main T6SS gene cluster and another T6SS-related gene cluster encoded elsewhere. The main cluster consists of the *imp* operon for the main structural T6SS (*tssA* to *tssM*) components and the *hcp* operon for genes coding for a puncturing device (*hcp* and *vgrG1*), effectors (*tae* and *tde1*), and the associated genes. The orphan *vgrG2* auxiliary operon harbors the *tde2* effector gene and the associated genes. Two of the secreted T6SS effectors, Tde1 and Tde2, are nucleases, and the remaining Tae is a putative peptidoglycan (PG) amidase. Tde1 and Tde2 are the main players in interbacterial competition with their nuclease activity, and deletion of both effectors eliminates all of the detectable killing activity to susceptible A. tumefaciens siblings *in planta* (11) or distantly related Escherichia coli *in vitro* (12). However, the level of antibacterial activity is relatively modest (about 0.5 to 1 log_10_) and far behind other T6SS-containing bacteria such as Vibrio cholera and P. aeruginosa (>3-log_10_ CFU inhibition of E. coli) (13, 14), although P. aeruginosa is not active against E. coli unless provoked, known as tit for tat (15).

Despite extensive studies of diverse functions of T6SS antibacterial effectors in a wide range of bacterial species, the rationale underlying different magnitudes of T6SS-dependent killing remains unknown. In this study, we first address whether A. tumefaciens T6SS killing activity can be enhanced and what are the conditions and factors required to trigger the full power of T6SS in A. tumefaciens. We demonstrated that the ability of the T6SS of A. tumefaciens to kill is increased to eliminate a large proportion of recipient target cells via carbon starvation or recipient cell wall PG modification. This led to the discovery of the new role for a highly conserved T6SS effector, Tae, a putative PG amidase. Under the condition allowing the growth of recipient cells, Tae but not Tde was the main player required to maintain competitiveness in a mixed population. Unlike Tde1 and Tde2, which are only found in certain strains, Tae is conserved in all sequenced A. tumefaciens T6SS-positive (T6SS^+^) genomes (9). This provides a new insight that some T6SS effectors, such as Tae, with weak phenotypes in laboratory conditions, could be overlooked and important for the bacterial species to maintain competitiveness among other bacteria.

## RESULTS

### Carbon starvation and PG disturbance in recipient cells enhance the T6SS-dependent killing outcome of A. tumefaciens.

**FIG 1:**
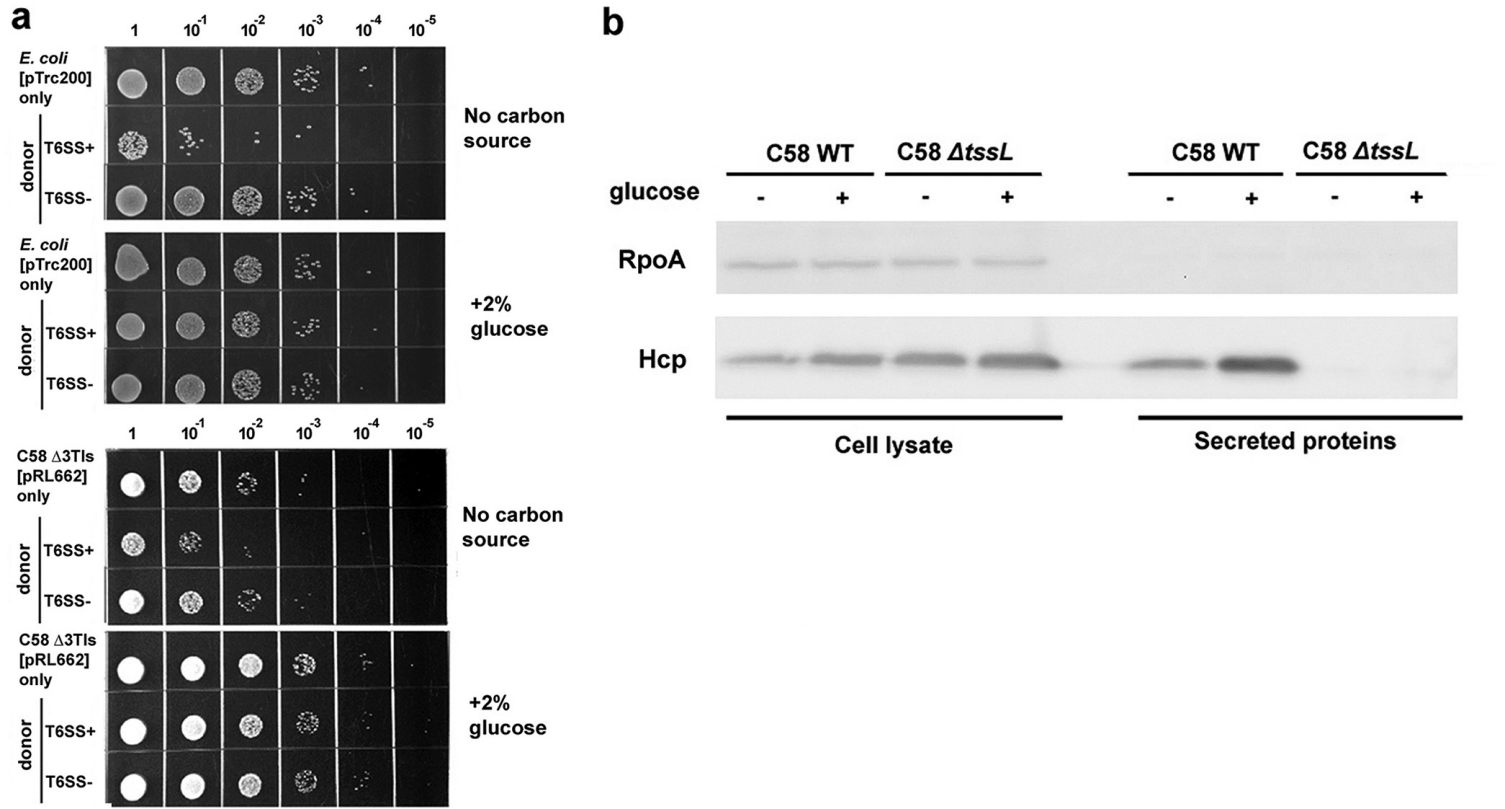
Carbon starvation leads to enhanced T6SS-dependent susceptibility in E. coli. (a) Recovery of recipient cells (E. coli or T6SS-susceptible mutant A. tumefaciens Δ3TIs) harboring pTrc200 after coincubation with A. tumefaciens with (T6SS^+^, i.e., WT) or without (T6SS^−^, i.e., Δ*tssL*) active T6SS on AK medium agar plate (no carbon source) or supplemented with glucose (+2% glucose) at a ratio of 30:1 (donor to recipient). The recovery of recipient cells on AK medium-starved condition (no carbon source) was significantly lower than under the glucose-fed condition. (b) Hcp secretion assay. Immunoblots using anti-RpoA and anti-Hcp antibodies for detecting Hcp and RNA polymerase subunit alpha (RpoA), respectively, in both cellular and secreted fractions of A. tumefaciens WT and Δ*tssL* grown in AK medium with or without glucose for 6 h. RpoA was a loading and nonsecreted protein control. Representative results of at least two independent experiments are shown.

A previous study showed that A. tumefaciens intraspecies killing activity or outcome could be observed only in an *in planta* assay but not on the *in vitro* acidic AB-MES (pH 5.5) agar plate (11), a minimal medium with glucose optimized for A. tumefaciens growth and virulence induction. Thus, we proposed that an environmental signal inside the plant may potentiate or activate A. tumefaciens T6SS activity or the *in vitro* growth condition may suppress A. tumefaciens T6SS-mediated killing activity or outcome. After testing various conditions (such as different carbon sources and additional plant apoplastic fluid), we discovered that A. tumefaciens could kill its susceptible sibling Δ3TIs (lacking all effector and cognate immunity genes) *in vitro* by coincubation in Murashige and Skoog agar medium, a commonly used plant culture medium lacking a carbon source. Further addition of apoplast fluid on Murashige and Skoog agar did not enhance the killing effect. The same killing ability between A. tumefaciens siblings could be also observed by removing glucose in cocultured AB-MES (pH 5.5) agar plates, which also further enhanced its T6SS killing of E. coli (see Fig. S1a and b in the supplemental material). Supplement of sucrose or glycerol instead of glucose also caused the full or partial suppression of this T6SS killing outcome, which suggests enhanced T6SS killing during carbon depletion. We further established an optimized acidic minimal medium called *Agrobacterium* kill-triggering (AK) medium (Fig. 1a) (16, 17). With AK medium, the killing activity was greatly increased, with ∼2-log_10_ inhibition of E. coli and ∼1.5-log_10_ inhibition of Δ3TI sibling cells (Fig. 1a). The killing activity can also be suppressed to the basal level of <0.5 log_10_ by supplementing glucose (i.e., AKG medium) (Fig. 1a). The enhanced T6SS killing outcome was not caused by increased T6SS secretion activity because the secretion levels of T6SS secretion hallmark Hcp were not elevated and instead were slightly reduced in the AK medium compared with AKG (Fig. 1b). The results suggest that the enhanced killing outcome could be caused by factors beyond T6SS secretion activity under the carbon depletion growth condition.

**FIG 2:**
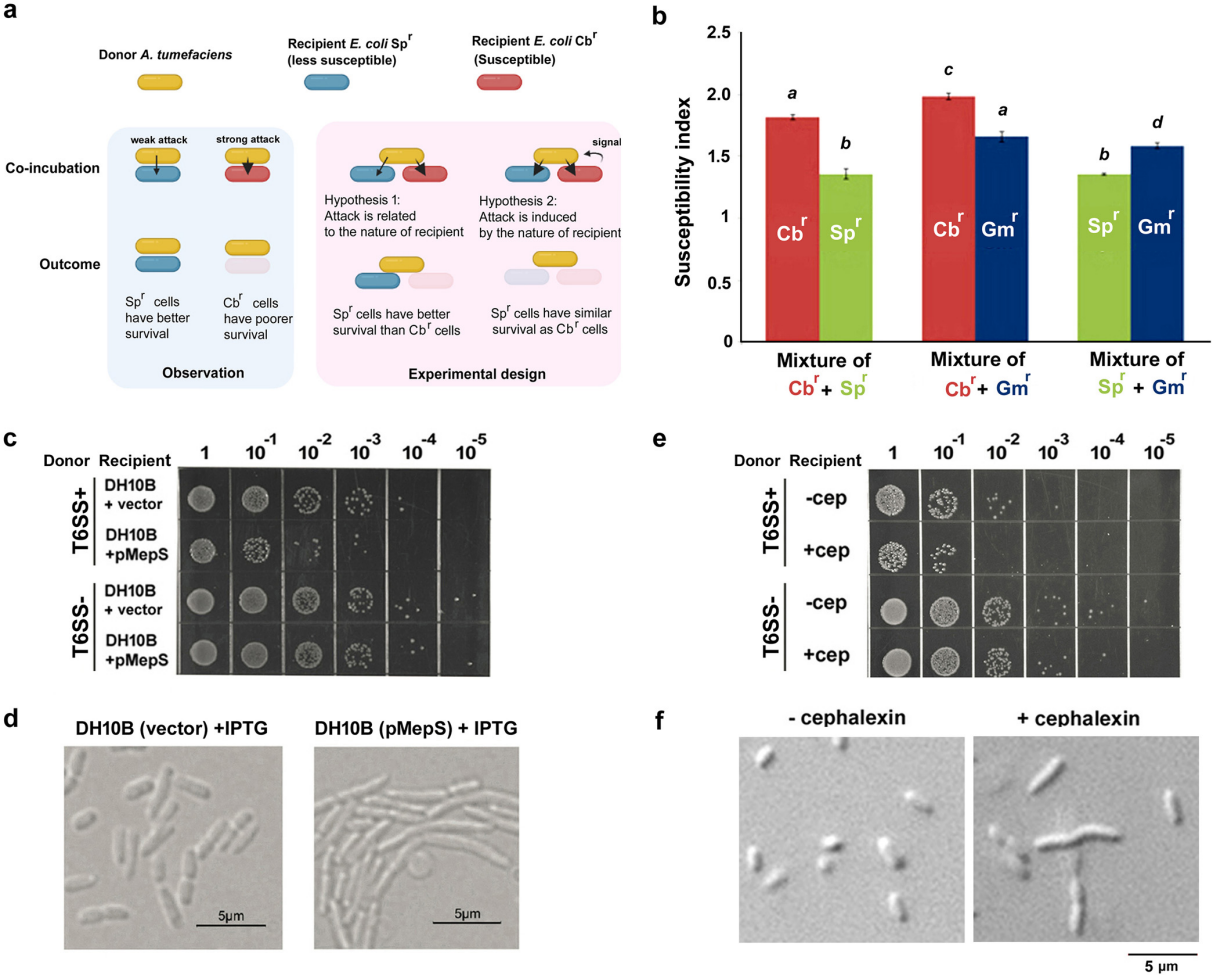
Peptidoglycan-related modifications in E. coli recipient cells lead to enhanced T6SS-dependent susceptibility. (a) Schematic diagram of the experimental design to study the rationale of the differential susceptibility of E. coli cells harboring different antibiotic-resistant genes. (b) T6SS-dependent susceptibility of E. coli DH10B recipient cells harboring different antibiotic-resistant genes (Cb^r^, carbenicillin resistant; Sp^r^, spectinomycin resistant; Gm^r^, gentamicin resistant). As demonstrated in panel a, two types of recipient cells were coincubated with A. tumefaciens with (i.e., WT) or without (i.e., Δ*tssL*) active T6SS on AK medium agar plates. E. coli harboring the carbenicillin-resistant gene was generally more susceptible than E. coli harboring spectinomycin- or gentamicin-resistant genes. Representative data with three biological replicates of two independent experiments are shown, and the samples are grouped by one-way analysis of variance (ANOVA) with a significant *P* value of <0.05. T6SS-dependent susceptibility index (SI) (17) was designated the logarithm-recovered CFU of that attacked by Δ*tssL* subtracted by that attacked by WT C58. The higher SI value indicates stronger A. tumefaciens T6SS killing. (c) Recovery of E. coli DH10B harboring vector (pTrc200) or *mepS*-expressing plasmid (pMepS) after coincubation with A. tumefaciens strain C58 with (T6SS^+^, i.e., WT) or without (T6SS^−^, i.e., Δ*tssL*) active T6SS on an AKG medium agar plate with IPTG. Recovery of DH10B overexpressing *mepS* (pMepS) was significantly lower than DH10B harboring vector (pTrc200). (d) Cell morphology of E. coli DH10B strains after growing in LB medium with or without IPTG for 4 h. (e) Recovery of cephalexin (3 μg/ml)-pretreated E. coli DH10B harboring pTrc200 after coincubation with A. tumefaciens with (T6SS^+^, i.e., WT) or without (T6SS^−^, i.e., Δ*tssL*) active T6SS on AKG agar. (f) Cell morphology of E. coli DH10B after treating with cephalexin (3 μg/ml) at log phase for 3 h. Representative results of at least two independent experiments are shown.

Recipient cell factors affecting contact-dependent growth inhibition were previously documented in contact-dependent inhibition (CDI) and T6SS (17, 18). Accidentally and repetitively, we found that T6SS susceptibility was generally higher if E. coli recipient cells carried a β-lactamase-expressing plasmid to confer resistance to β-lactams (ampicillin/carbenicillin [Ap/Cb]) rather than a plasmid expressing a spectinomycin or a gentamicin resistance gene (Fig. S1c). The observation was not likely due to the reported “tit-for-tat” activation (15) because the recipient E. coli strain has no T6SS. We then designed an experiment to verify whether the observation was due to (i) higher susceptibility of the Ap/Cb-resistant cells or (ii) a stronger attack triggered by the Ap/Cb-resistant cells (Fig. 2a). We mixed two populations of recipient cells expressing either β-lactamase (pBluescript for carbenicillin [Cb^r^]) or an aminoglycoside resistance gene (pTrc200 for spectinomycin [Sp^r^]/pRL662 for gentamicin [Gm^r^]) (19) and then checked the differences in recipient cell recovery. When mixing with Cb^r^ cells, the susceptibility of Sp^r^ cells remained the same and was slightly enhanced for Gm^r^ cells. The Cb^r^ cells were consistently more susceptible than Gm^r^ or Sp^r^ cells (Fig. 2b). Thus, Cb^r^ cells may not universally reinforce A. tumefaciens to exert stronger killing activity to Gm^r^ or Sp^r^ cells. β-Lactamase can induce changes in the cell wall PG composition (20), which could be a reason for the higher susceptibility of the Cb^r^ recipient cells. To further confirm this hypothesis, E. coli cells overexpressing a PG dd-endopeptidase gene, *mepS*, were used as recipient cells in the killing assay. MepS controls cell wall synthesis in E. coli by cleaving the d-Ala-*meso*-diaminopimelic acid (mDAP) cross-links in the PG layers (21). Overexpressing *mepS* leads to an enhanced killing outcome (Fig. 2c) and cell overelongation (Fig. 2d). The overelongation was similar to the observation when a subinhibitory amount of the PG-targeting antibiotic cephalexin is applied to E. coli cells (22). We then pretreated recipient cells with cephalexin, and cell elongation (Fig. 2f) and enhanced susceptibility (Fig. 2e) were also observed. Endogenous expression of a β-lactamase or PG dd-endopeptidase gene *mepS* and exogenous application of cephalexin have a common effect in changing the PG composition/balance. Both environmental factors (depletion of carbon source) and recipient factors (PG disturbance) are able to enhance the T6SS-dependent killing outcome, which have provided clues to how A. tumefaciens regulates its T6SS during interbacterial competition.

### Overexpression of Tae in E. coli led to cell elongation and enhanced T6SS-dependent susceptibility.

**FIG 3:**
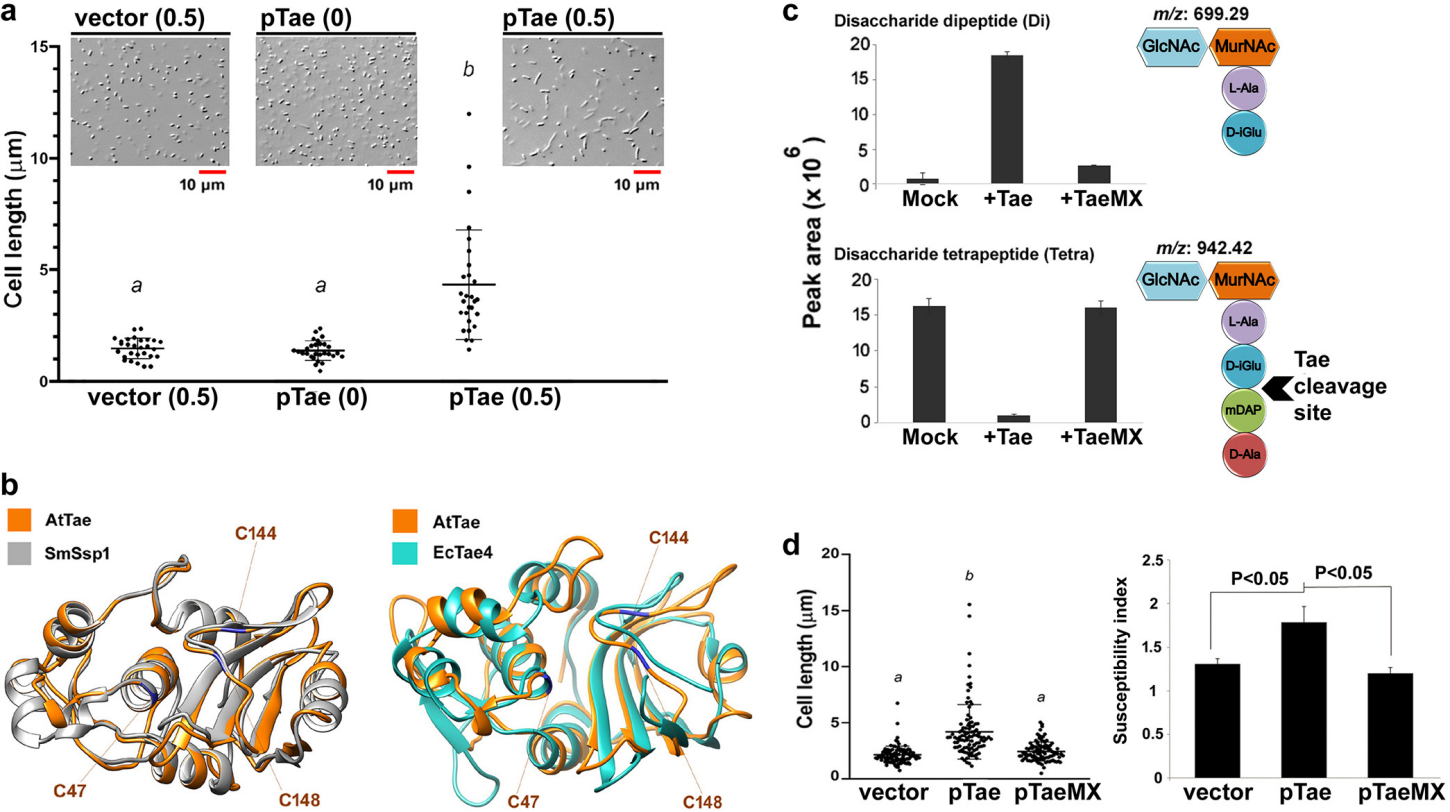
AtTae is a peptidoglycan amidase causing cell elongation and enhances T6SS susceptibility when ectopically expressed in E. coli. (a) E. coli DH10B harboring vector control (pTrc200) or Tae-expressing vector (pTae) was induced by IPTG (0.5 mM) in LB medium for 4 h. The cells were observed under a light microscope, and the longitudinal cell lengths was recorded. A representative experiment of three independent experiments is shown (*n* = 28; one-way ANOVA; *P* < 0.001). (b) Three-dimensional structure comparison between AtTae (orange; PDB ID 6IJF) and SmSsp1 (grey; PDB ID 4BI3) or EcTae4 (green; PDB ID 4HFL) based on the jFATCAT_rigid algorithm (43) and drawn by UCSF Chimera (44). Residues substituted in the mutant TaeMX (AtTae residues C47, C144, and C148) are highlighted. (c) UPLC-MS analysis of E. coli peptidoglycan after digestion AtTae. Peak area of disaccharide dipeptide (Di) product (*m/z*, 699.29; apex RT, 3.0 min) was massively enriched after incubation with WT Tae, but the peak area of disaccharide tetrapeptide (Tetra; *m/z*, 942.42; apex RT, 2.96 min) was significantly reduced. The pattern was not observed when peptidoglycan was inoculated with nonfunctional TaeMX protein. This indicates that Tae targets and cleaves the bonds d-Glu and mDAP in peptidoglycan. Data are means ± standard deviations (SD) from of three technical replicates. Similar results were obtained from two independent experiments. (d) Expression of the *tae* mutant-producing TaeMX (C47A, C144A, and C148A) did not promote a significant difference in cell elongation in E. coli from that of the WT Tae. Cell length was the mean of three independent experiments (*n* = 100; one-way ANOVA; *P* < 0.001). Susceptibility index (SI) of IPTG-induced E. coli DH10B cell-expressing Tae is enhanced. Recipient E. coli cells harboring vector control (pTrc200), WT Tae (pTae), and TaeMX (pTaeMX) were coincubated with donor cells A. tumefaciens WT and Δ*tssL* mutant (donor-to-recipient ratio, 30:1) on AKG agar. T6SS-dependent SI was designated the logarithm-recovered CFU of that attacked by Δ*tssL* subtracted by that attacked by WT C58. The higher SI value indicates stronger A. tumefaciens T6SS killing. Data are means ± SD from three biological replicates by Student’s *t* test with a significant *P* value of < 0.05. Representative data of three independent experiments are shown.

Like MepS, many T6SS effectors possess endopeptidase activity to the PG layers but with different amide bond targets and cytotoxicity (23). For example, Pseudomonas aeruginosa Tse1 (24) and Serratia marcescens Ssp2 (25) belong to the Tae4 family attacking the bonds mDAP-d-Glu, whereas Tse1 cleaves mGlu-d-Ala in PGs (23). From the molecular structure, A. tumefaciens Tae (AtTae) can be classified as the Tae4-type (24, 25) PG amidases such as SmSsp1 and SmSsp2 of Serratia marcescens (25, 26) and EcTae4 of Enterobacter cloacae (27). With the recently resolved three-dimensional structure of AtTae (28), our comparison further reveals that AtTae is better superimposed with SmSsp1 (39.39% identity) than EcTae4 (15.79% identity) (29). This finding is consistent with the notion that SmSsp1, a relatively close homolog of AtTae (Fig. S2), only exhibits a weak killing phenotype, and similarly, no antibacterial activity of AtTae could be detected in our previous experimental setups for interbacterial competition (11, 12). However, EcTae4 and SmSsp2 belonging to the same phylogenetic group but more distant from AtTae exhibit prominent antibacterial phenotypes (5, 25–27). Among these Tae4 amidases, three cysteine residues, C47, C144, and C148, are highly conserved (27) (Fig. S2 and Fig. 3b). Since the biochemical activity of AtTae was not demonstrated, His-tagged Tae was overexpressed in E. coli and purified for PG digestion assay (Fig. S3a). The data showed that AtTae was able to cleave the bonds between d-Glu and mDAP in PGs isolated from E. coli (Fig. 3c and Fig. S3b). Substitution of the conserved three cysteine residues to alanine (TaeMX) abolished the formation of a homodimer and a potential internal secondary structure as well as PG amidase activity (Fig. S3a). Based on the structural comparison and Western blot analysis (Fig. 3b and Fig. S3a), C47 can be assigned as catalytic residue, whereas C144 and C148 can be assigned as structural disulfide bond-forming residues. The formation of homodimer is a minority species which is due to the weak interaction of C47. In conclusion, we demonstrate that AtTae exhibits Tae4 family amidase activity and is closely related to SmSsp1 but phylogenetically separate from SmSsp2 (26) and EcTae4 (27).

T6SS PG amidases are expected to inject into the recipient cell wall without the requirement of a signal peptide. AtTae and SmSsp1 do not exhibit potent antibacterial activity in the interbacterial competition context, but they are able to inhibit bacterial cell growth when they are expressed with fusion to Sec-dependent signal peptide in E. coli (11, 26). How these PG toxins reach their periplasmic destination once injected into the recipient cells remains unclear. A recent study of Vibrio cholerae VgrG3 provided evidence that a linker domain between the gp27/gp5 domain and C-terminal extension of VgrG3 is required for VgrG3 trafficking from the cytoplasm to periplasm (30). As we can observe of the cell elongation phenotype when E. coli cells overproduce AtTae (native form without fusion to Sec signal peptide) (Fig. 3a), we suggest that AtTae may traffic to the periplasm like VgrG3 but in a less effective manner in recipient cells. Importantly, E. coli cells overexpressing AtTae were more susceptible to *Agrobacterium* T6SS killing, as demonstrated with higher T6SS-dependent susceptibility by calculating, the CFU difference of recovered E. coli coincubated with the wild type (WT) and Δ*tssL* (Fig. 3d). Substitution of the conserved cysteine residues (Fig. S2) to alanine (TaeMX) abolished the formation of homodimer and a potential internal secondary structure (Fig. S3a) as well as the Tae overexpression phenotypes (Fig. 3d). In conclusion, ectopic expression of AtTae in E. coli cells caused both cell elongation and enhanced T6SS-killing susceptibility dependent on PG amidase activity.

### Differential morphological changes of E. coli recipient cells on receiving different T6SS effectors from A. tumefaciens.

Phenocopy of E. coli cells with ectopic expression of AtTae in MepS-overexpressing or cephalexin-treated E. coli cells led us to hypothesize that T6SS-injected Tae toxin interferes in PG biogenesis of recipient cells to lead to cell elongation and enhanced susceptibility. Thus, we next examined whether cell elongation phenotypes observed in E. coli expressing AtTae could be observed in an interbacterial competition event. Because the Tae-related phenotype could be massively masked by the presence of two strong bactericidal Tde effectors, we generated an A. tumefaciens C58 mutant strain with chromosomal substitutions of the conserved cysteine residues (C47A, C144A, and C148A) in both the WT (i.e., C58::*taemx*) and Δ*2tdei* mutant (deletion of two *tde1*-*tdi1* and *tde2*-*tdi2* effector-immunity pairs) (i.e., Δ*2tdei::taemx*). Tae amidase activity was not essential for T6SS assembly because C58::*taemx* remained active in Hcp and Tae secretion (Fig. S4a). However, use of the Δ*2tdei* mutant lacking both Tde1 and Tde2 effectors largely attenuated the overall T6SS assembly and secretion activity (12). To compensate for this, we transformed a plasmid (pEML4286 [Table S1]) expressing the Tde1 variant (H190A D193A, Tde1M) with loss of DNase activity (11) and its associated genes required for loading Tde1M onto the VgrG1 spike for activating T6SS assembly (31) into Δ*2tdei* to allow for Hcp and Tae secretion without the Tde toxicity (Fig. S5a).

**FIG 4:**
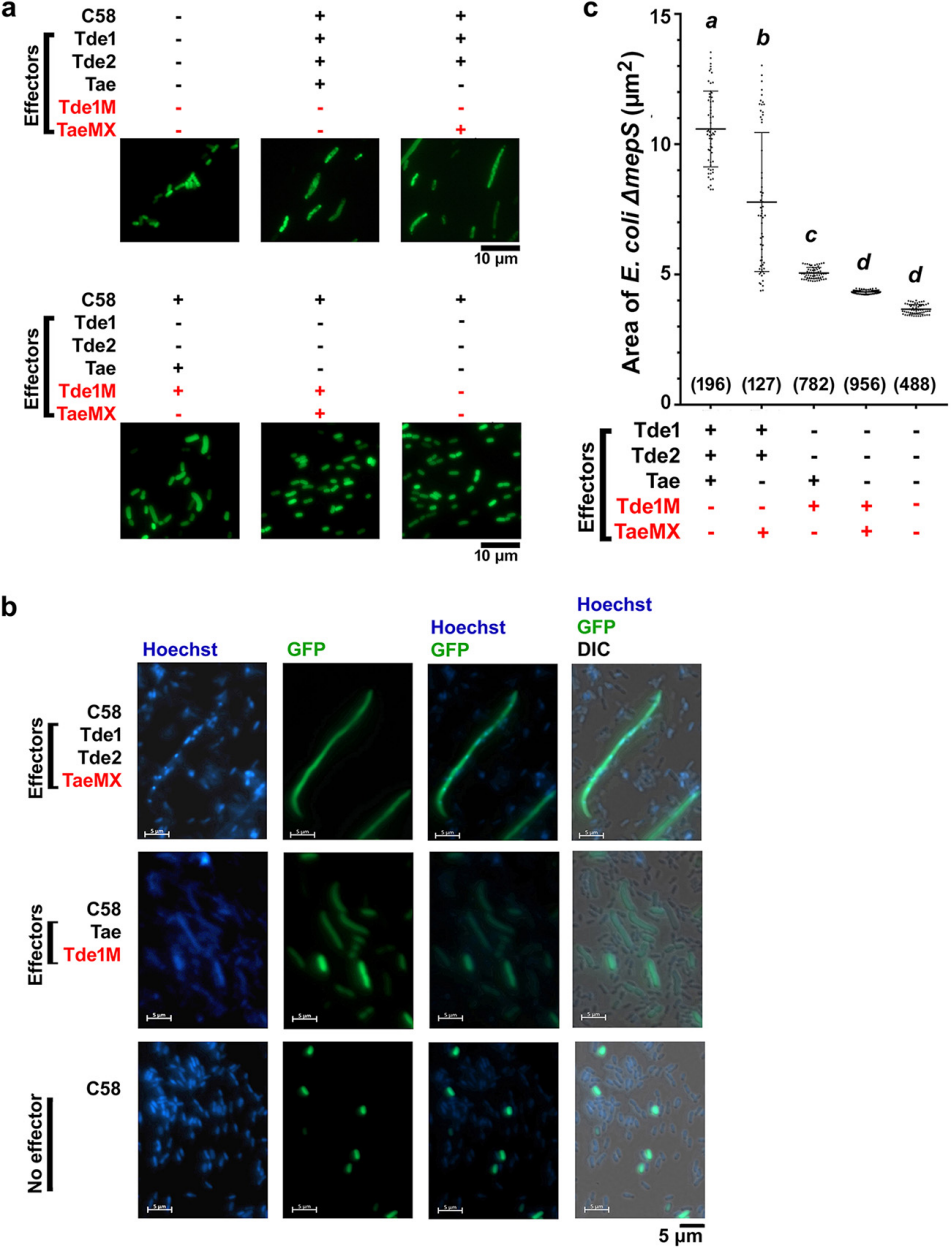
A. tumefaciens C58 deploys different effectors, leading to changes in the morphology of recipient E. coli cells. (a) E. coli Δ*mepS* expressing pRL-GFP(S65T) was inoculated with C58 with different combinations of T6SS effectors. Donor cells (C58) and recipient cells (Δ*mepS*) were mixed in a 9:1 ratio and spotted on 523 agar plate for 3 h at 28°C. After inoculation, cells were observed under a fluorescence microscope at ×100 magnification. The A. tumefaciens strains used were WT C58 containing all functional effectors (Tde1^+^/Tde2^+^/Tae^+^), *taemx* carrying functional Tde effectors and a nonfunctional Tae (Tde1^+^/Tde2^+^/TaeMX^+^), Δ*2tdei* plus pEML4286 carrying a functional Tae and a nonfunctional Tde1 (Tae^+^/Tde1M^+^), Δ*2tdei::taemx* plus pEML4286 carrying a nonfunctional Tae and a nonfunctional Tde1 (TaeMX^+^/Tde1M^+^), and Δ3TIs lacking any of the three effectors (no effectors). Δ*mepS* (GFP) not incubated with any A. tumefaciens donor (no donor cells) was used as a control. (b) Similar to the above, the cells were stained with Hoechst before observation under the microscope to visualize genetic materials inside the cells. On incubation with only the donor expressing effective Tde effectors (Tde1^+^/Tde2^+^/TaeMX^+^), Δ*mepS* cells showed heavy elongation and segmentation of genetic material, whereas donor cells expressing Tae (Tae^+^/Tde1M^+^) led to the enlargement of cells with intact genetic material. With donor lacking no effectors, Δ*mepS* cells remained uniform in cell size. The text in red indicates the nonfunctional effector in A. tumefaciens. (c) Cellular area of an individual Δ*mepS* cell as reflected from the GFP signals in the experiment of panel a was automatically measured by ImageJ and then manually confirmed. The text in red indicates the nonfunctional effector. The numbers in the brackets indicate the total number of cells counted, and the cell sizes of ±30 cells of the median are shown and grouped by one-way ANOVA with a significance value of *P* < 0.05.

E. coli Δ*mepS* expressing green fluorescent protein (GFP) was used as the recipient to validate the effect of Tae after coincubation with an A. tumefaciens donor. E. coli Δ*mepS* is a PG-impaired strain and was preferred because of its uniform and short cell length relative to WT BW25113 (32). Under a fluorescence microscope, most of the GFP-expressing E. coli cells were enlarged after coincubation with A. tumefaciens expressing a functional Tae alone (Tae^+^/Tde1M^+^) (Fig. 4a). In contrast, most of the recipient cells remained with a similar short rod shape like the control (E. coli Δ*mepS* alone) when coincubated with A. tumefaciens donors expressing only nonfunctional Tae (TaeMX^+^/Tde1M^+^) or no effectors (Δ3TIs). Unexpectedly, the recipient cells exhibited massive elongation when coincubated with the A. tumefaciens donor that produced functional Tde1 and Tde2 effectors (Tde1^+^/Tde2^+^/Tae^+^ or Tde1^+^/Tde2^+^/TaeMX^+^) (Fig. 4a) regardless of the presence of Tae. Although Tde and Tae transformed the recipient cells differently, both led to cell enlargement as reflected by the cell area (Fig. 4c). Condensed DNA was also observed in elongated recipient cells only when coincubated with an A. tumefaciens donor that produces functional Tde effectors (Tde1^+^/Tde2^+^/Tae^+^ or Tde1^+^/Tde2^+^/TaeMX^+^) (Fig. 4b and Fig. S6), a similar observation reported in other T6SS DNase toxins (33). No condensed DNA was detected in elongated E. coli Δ*mepS* cells induced by coculture with A. tumefaciens expressing functional Tae alone (Tae^+^/Tde1M^+^). These results suggest that translocation of Tde DNase effectors into E. coli cells results in increased cell length and DNA condensation, whereas Tae translocation caused a modest increase of E. coli cell size with no effect on DNA condensation. Of note, cell morphology differed between Tae overexpression (Fig. 3a) and direct translocation of the effector from the donor A. tumefaciens, which could be due to the differences in the rate of translocation and E. coli strains used.

### AtTae suppresses the recovery of recipient cells and is important for A. tumefaciens to maintain competitiveness in a bacterial population.

In the context of interbacterial competition, Tae did not appear to demonstrate a significant role (Fig. S4b and Fig. S5a) compared with Tde effectors in our previous report (11). This may be related to strong interbacterial competition activity contributed by Tde and less understanding of the gene regulation mechanism in the *hcp* operon (Fig. S5) (12).

**FIG 5:**
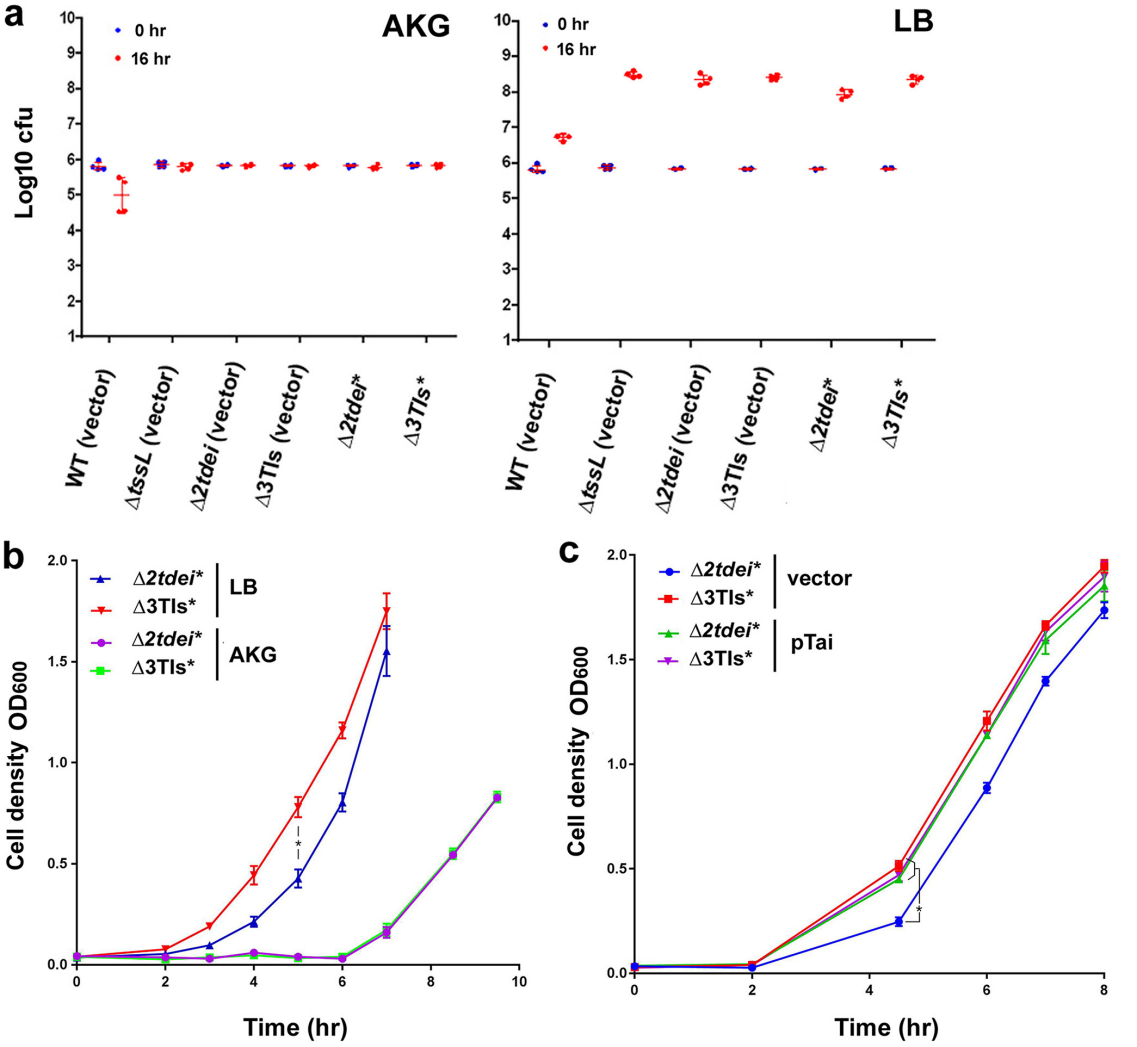
Tae-expressing A. tumefaciens retards the growth of recipient cells during coinoculation on rich medium. (a) CFU of recovered E. coli DH10B harboring pRL662 after coinoculation with strains of A. tumefaciens on AKG agar or LB agar at time 0 and after 16 h. The data are means ± SD from six biological replicates from three independent experiments. (b) Growth curve of E. coli DH10B harboring pRL662 after coinoculation with A. tumefaciens strain with only functional AtTae secretion (Δ2tdei*, Δ*2tdei* plus pEML4286) or no secretion of any effectors (Δ3TIs*, Δ3TIs plus pEML4286) (see Fig S5 in the supplemental material for details) on AKG agar or LB agar at a ratio of 30:1 (donor to recipient) for 16 h at 28°C. Data are means ± SD from three biological replicates. Representative data of three independent experiments are shown (*, *P* < 0.05). (c) Growth curve of DH10B harboring empty vector or derivative expressing Tae immunity protein (pTai) after coinoculation with A. tumefaciens strain Δ2tdei* or Δ3TIs* on LB agar at a ratio of 10:1 (donor to recipient) for 16 h at 28°C. Data are means ± SD from three biological replicates. Representative data of three independent experiments are shown (*, *P* < 0.05).

To address whether Tae plays a role in interbacterial competition, we monitored the recovery of E. coli recipient cells after A. tumefaciens-E. coli coincubation. The procedures were similar during the donor-recipient coincubation, but the coincubated bacterial suspension was normalized to the same optical density at 600 nm (OD_600_) followed by growth curve analysis in LB broth with supplement of antibiotics at 37°C to recover the E. coli growth while killing agrobacteria. We first compared the recovery times of WT, *taemx* (genomic *tae* loss-of-function mutant), and Δ*tssL* strains. We found a minor but repeatable faster recovery of the *taemx* strain than WT (Fig. S7), which suggests that Tae may have a role in suppressing the growth of recipient cells during coincubation. The recovery was much faster when the Δ*tssL* strain was used as the donor, which suggests a prominent killing effect of Tde effectors during coincubation. To remove the effect of Tde effectors, we used donors only having a functional Tae (Δ*2tdei* plus pEML4286, abbreviated as Δ2tdei*) or no effectors (Δ3TIs plus pEML4286, abbreviated as Δ3TIs*). The recovery time was longer when the Δ2tdei* strain was used as the donor with LB as the coincubation medium. The difference in recovery time was not observed when AKG medium was used as the coincubation medium (Fig. 5b). E. coli did not grow on AKG medium, but it can grow on LB agar, as reflected by the CFU recovery between 0 h and 16 h (Fig. 5a). The above findings suggest that Tae may be effective only when the recipient cells are in a growing stage. Tai (Atu4346) is the immunity protein of Tae (11) and has been shown to physically interact with Tae in a structural study (28). However, no physiological evidence was available for the interbacterial competition context. A *tai*-expressing plasmid (pTai) was transformed into the recipient cells to verify the protective role of Tai against Tae in growth inhibition after translocation. The inhibitory effect from Δ2tdei* was not distinguishable in recipient cells harboring pTai (Fig. 5c), so Tai is the Tae immunity protein, and the growth inhibition effect observed was specific to Tae.

**FIG 6:**
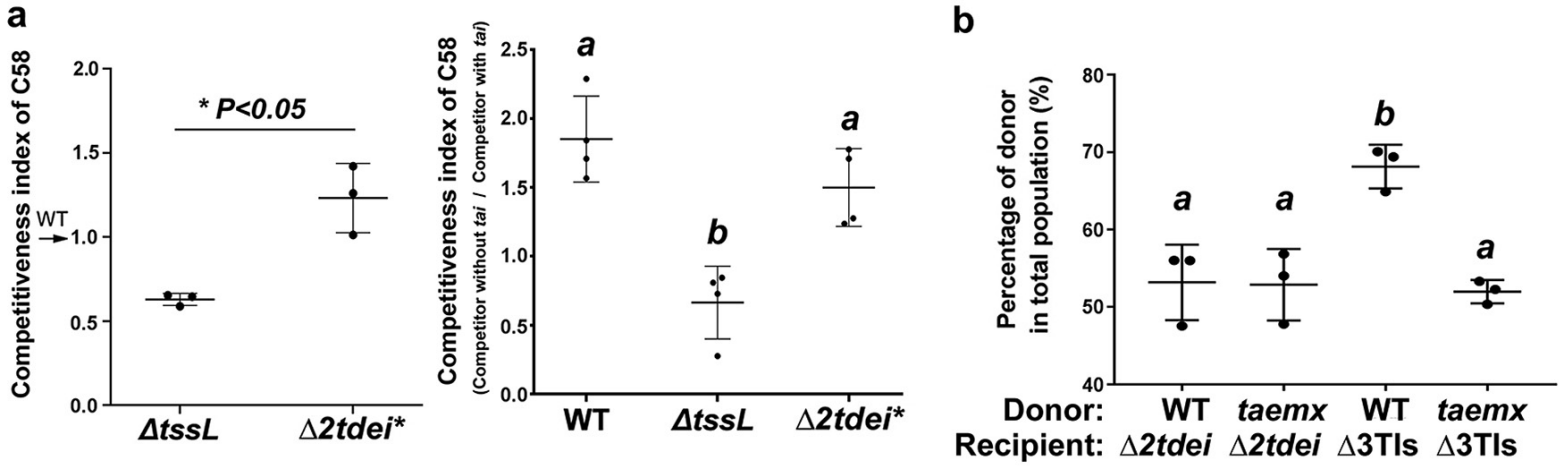
Tae-expressing A. tumefaciens shows better competitiveness in a bacterial population. (a) A. tumefaciens with Tae shows better competitiveness among E. coli cells. WT (WT plus pTrc200), Δ*tssL* (Δ*tssL* plus pTrc200), and a strain with only functional AtTae secretion (Δ2tdei*) of A. tumefaciens strains were each coinoculated with E. coli BW25113 at a 1:9 ratio on an LB agar plate for 16 h. The population of Δ*tssL* and Δ2tdei* cells among the E. coli cells was quantified by qPCR and represented as an index relative to the population of WT. Similarly, the A. tumefaciens strains were coincubated with BW25113 harboring a control vector (−*tai*, pRL662) or *tai*-expressing vector (+tai, pTai). The population of A. tumefaciens strains among the BW25113 cells without *tai* (*−tai*) was quantified by qPCR and represented as an index relative to the population of A. tumefaciens strains among the BW25113 cells with *tai* (*+tai*). The index (competitiveness index) is 1 if there is no advantage between two strains/conditions but >1 if there is an advantage and vice versa. Each dot represents an average of three technical replicates in an experiment. Data are means ± SD from at least three independent experiments. (b) The proportion of C58 donor cells with (WT) or without functional Tae (*taemx*) when coinoculated with recipient C58 cells with (Δ*2tdei*) or without (Δ3TIs) *tae*-*tai* toxin immunity pair. The cell proportion of donor cells in a mixed population was determined by qPCR with specific primers of *tde1* and 16S rRNA genes. The WT strain used is the WT *tae* gene recovered in Δ*tae* that underwent the same process in generating *taemx*. Each dot represents an average of three technical replicates in an experiment. Data are means ± SD from three independent experiments.

We next addressed the benefit of having Tae for *Agrobacterium* during interbacterial competition. The role of Tae may be more critical when A. tumefaciens cells are present as a minority in a bacterial population. The CFU recovery used for the classical T6SS-killing assay was not preferred because the survival rate cannot reflect the competitiveness of the donor cells when present as a minority. Thus, quantitative PCR was used to determine the relative abundance of agrobacterial cells in a mixed population directly and represented as an index (i.e., competitiveness index) to show the relative competitiveness of A. tumefaciens. Various A. tumefaciens strains were each mixed with E. coli at a 1:9 ratio on a LB agar plate for 16 h before quantitative PCR (qPCR) analysis. The relative abundance of the Tae-containing strain (Δ2tdei*) was similar to that of WT C58 (index, ∼1) but significantly higher than that without a functional T6SS (Δ*tssL*) (Fig. 6a). This competitiveness was lost if the opponent E. coli cells harbored pTai, which provided protection from Tae inhibition. The ability to kill opponents may not necessarily render an increased attacker population. To detect whether having Tae also provides an advantage among siblings, A. tumefaciens donor and recipient cells were mixed at a ratio of 1:1 on 523 medium optimized for A. tumefaciens growth. After coincubation, the population distribution was similar between recipient and donor cells (∼50%) when the recipient cells had a *tae*-*tai* pair (Δ*2tdei*) or donor cells had a nonfunctional *tae* gene (*taemx*). In contrast, the population of donor cells with a functional *tae* gene (WT) was significantly increased when the recipient cells lacked a *tae*-*tai* gene pair (Δ3TIs) (Fig. 6b). The findings reveal that AtTae is important in maintaining a competitive growth advantage for A. tumefaciens attackers in competing with E. coli or its own A. tumefaciens sibling cells.

## DISCUSSION

In this study, we discovered that variations in environmental and biological factors can affect the T6SS killing outcome. Depletion of a carbon source in the coincubation environment could render the A. tumefaciens T6SS-mediated intraspecies interbacterial competition phenotype that could not be observed in nutrient-rich growth conditions (Fig. 1a; see also Fig. S1a in the supplemental material). Also, the disturbance of the recipient cell wall (PG) could enhance the susceptibility (Fig. 2), and such disturbance could be subtle and not severely impact the recipient cell physiology (Fig. 2a). The observation led us to review the roles of *Agrobacterium* T6SS effectors, especially Tae, whose interbacterial competition activity was not demonstrated. In this study, we showed that Tae exhibits PG amidase activity and function to inhibit recipient cell growth in the interbacterial competition context. Importantly, Tae may not kill like Tde1 and Tde2 DNase effectors, but it is important for the growth advantage of A. tumefaciens in competing with growing E. coli or its own A. tumefaciens sibling cells.

Tde DNase effectors induced cell elongation and DNA condensation of recipient E. coli cells, whereas Tae caused a modest increase in cell size without DNA condensation (Fig. 4b and Fig. S6). The nuclease-dependent cell elongation phenotype is not a precedent for Tde; indeed, a previous study expressing P. aeruginosa Tse7 nuclease also showed increased cell length of E. coli cells (34). The increased cell elongation may be induced by an SOS response triggered by DNA damage (34) upon Tde DNase cleavage. Tae-dependent cell elongation was also observed when Tae was ectopically expressed in E. coli and was likely a growth inhibition effect caused by cleavage of PG bonds. The effect also phenocopies MepS-overexpressing or cephalexin-treated E. coli cells. A similar cell elongation phenotype was observed in an S. marcescens Δ*rap2a* mutant with deletion of the SmSsp1 immunity gene, and complementation of the *rap2a* gene in Δ*rap2a* could convert the cell shape to the WT (26).

Although AtTae, SmSsp1, SmSsp2, and EcTae4 are all classified to the Tae4 family that cleave the bonds between d-Glu and mDAP of PG based on *in vitro* tests (26, 27), our sequence and structural comparisons revealed that AtTae is more closely related to SmSsp1 but more distinct from EcTae4 and SmSsp2 (Fig. 3b and Fig. S2). Such a difference may be the cause to differentiate the observed antibacterial phenotype between the two subclasses (AtTae and SmSsp1 versus SmSsp2 and EcTae4). We noted that EcTae4 contains an additional beta-sheet loop that is absent in both AtTae and SmSsp1 (Fig. 3b) in addition to dispersed dissimilarity of primary amino acid sequences between these two groups of Tae proteins (Fig. S2). Future work to carry out domain swapping or site-directed mutagenesis may provide insights into the discrepancy of antibacterial phenotypes.

**FIG 7:**
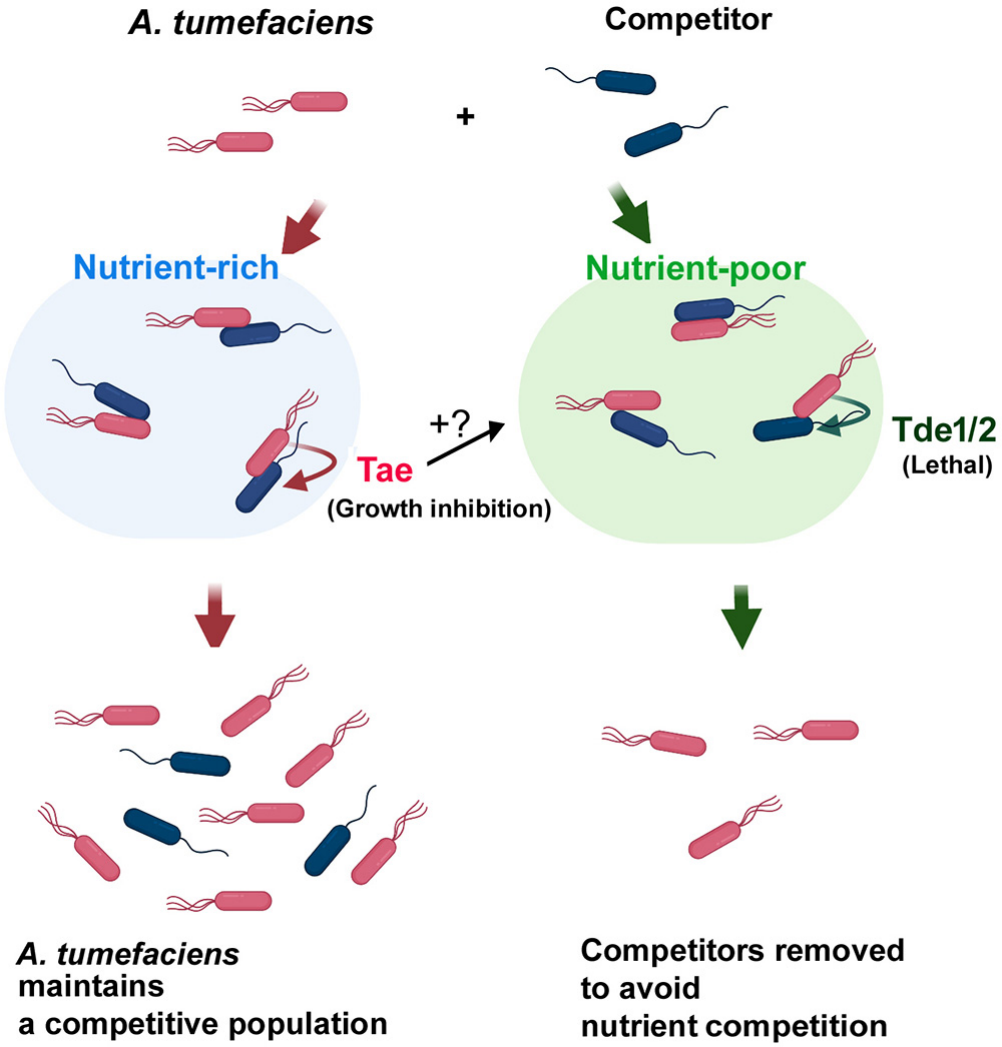
Proposed antibacterial strategy of A. tumefaciens to compete with bacterial competitors. A. tumefaciens C58 deploys two types of effectors. When nutrients are abundant, Tae effector inhibits the growth of recipient cells to maintain a competitive proportion in a population. When nutrients are deficient, Tde effectors are dominant over Tae, and the competitors are eliminated to avoid further nutrient competition. The antibacterial strategy may be important for A. tumefaciens to compete with other bacteria in different habitats. Tae may be able to assist the efficiency of Tde effectors as observed in the Tae-expressing E. coli cells, but further studies are required to prove this hypothesis.

Fast-growing opponents such as E. coli could have a growth advantage over A. tumefaciens when nutrients are available, and in this scenario, although Tde1 and Tde2 are potent toxins, they may not be effective enough to stop the growth of opponents. This could lead to a gradual reduction of A. tumefaciens proportion in a bacterial population. Indeed, having only Tae as a sole T6SS toxin was found sufficiently effective to maintain a higher proportion of A. tumefaciens in a mixed bacterial population (Fig. 6). Tde toxins may be preferable in conditions with very limited resources (e.g., carbon starvation) to kill recipient cells for reducing competition of nutrients. A recent report also indicated that accepting the DNA fragments from damaged recipient cells is not necessarily beneficial to the attacker cells (35). However, when nutrients are available, A. tumefaciens may use Tae to effectively limit the growth of opponents in order to maintain a respectable presence among other fast-growing bacteria species coexisting in the same ecological niche. These two strategies (Fig. 7) could be equally important for A. tumefaciens to adapt in different scenarios. A. tumefaciens, a soil inhabitant, could encounter various growth environments ranging from rhizosphere (a nutrient-rich environment) to apoplast inside plants (an acidic nutrient-poor environment) (36–38). Thus, agrobacteria equipped with both Tae and other variable effectors could be a versatile strategy for competing in variable living habitats from free living in soil to rhizosphere and crown gall. How Tae and Tde effectors cooperate in various conditions to achieve the best advantage among other bacterial species remains unknown, but the increased susceptibility in Tae-overexpressed E. coli (Fig. 3d) could give a clue that Tae may assist Tde effectors when the environment becomes unfavorable. Further exploration of the role of the effectors in the natural habitats and opponents should provide a better picture of the role of T6SS in microbial ecology.

## MATERIALS AND METHODS

### Bacterial strains and plasmids.

Information on strains and plasmids created in this study is listed in Table S1 in the supplemental material. E. coli strains were grown in Luria-Bertani (LB) medium at 37°C supplemented as appropriate with antibiotics, 25 μg · ml^−1^ gentamicin or 100 μg · ml^−1^ spectinomycin. A. tumefaciens strains were grown in 523 medium (39) at 28°C supplemented as appropriate with 25 μg · ml^−1^ gentamicin or 100 μg · ml^−1^ spectinomycin.

### Interbacterial competition assays.

A. tumefaciens strains as donors were grown in 523 medium with appropriate antibiotics at 28°C for overnight and harvested by centrifugation. The recipient E. coli cells harboring plasmids conferring selectable antibiotic resistance were grown in LB medium with appropriate antibiotics at 37°C overnight and harvested by centrifugation. The harvested cells were washed with 0.9% saline and resuspended in 0.9% saline. Donor cell density was adjusted to OD_600_ of 3, and recipient cell density was adjusted to 0.1 or 0.3. Donor and recipient were mixed at 1:1 (vol/vol) to make the cell density ratio 30:1 or 10:1 or indicated otherwise. The mixture was spotted on an agar plate (wt/vol; 1.5%) with the indicated media and incubated for 16 h at 25°C. After coincubation, the spot was resuspended in 0.9% saline, serial diluted, and spotted or plated on an LB agar plate with appropriate antibiotics for recipient cell selection. The CFU were counted, and the susceptibility index (SI) (17) was defined as the logarithm of the recovered cells cocultured with Δ*tssL* and subtracted by the recovered cells cocultured with another donor. The higher SI value indicates more susceptibility of the recipient cells to the T6SS-dependent attack. The AK minimal medium (17.2 mM K_2_HPO_4_, 8.3 mM NaH_2_PO_4_, 18.7 mM NH_4_Cl, 2 mM KCl, 50 mM morpholineethanesulfonic acid [MES], and 2% glucose [wt/vol], pH 5.5) was derived from AB-MES (40) without CaCl_2_, FeSO_4_, and MgSO_4_ ions. AK medium was preferred over AB-MES medium in the interbacterial competition assays because it is easier to prepare with on par or with a slightly better outcome.

### Secretion assay.

Secretion from liquid culture was assayed in 523 or AKG medium for 4 to 6 h at 25°C as previously described (41) with modification. In brief, 1 ml cells grown overnight in 523 medium adjusted at an OD_600_ of 1 were harvested by centrifugation at 10,000 × *g* for 5 min. The resulting pellets are cellular fractions, and the supernatant (secreted fraction) was mixed with a final concentration of sodium deoxycholate (0.03%) and trichloroacetic acid (15%). The protein precipitation was performed at −20°C for 1 day, and proteins were collected by centrifugation at 21,130 × *g* for 10 min at 4°C. The supernatant was removed completely, the precipitated secreted proteins were resuspended in 50 μl of 2× SDS loading dye, and the cellular fractions were resuspended in 2× SDS loading dye for SDS-PAGE and Western blot analysis. Western blot analysis was performed as described (10).

### Tae enzymatic activity analysis.

Each of the Tae-expressing vectors (pTae-HA-His and pTaeMX-HA-His) were transformed into E. coli BL21 for protein expression. The cells at mid-log phase (range of OD_600_, 0.4 to 0.6) were induced by 1 mM isopropyl-β-d-thiogalactopyranoside (IPTG) for 6 h at 25°C, harvested, and ruptured by sonication. The six-His-tagged Tae proteins were purified with Ni-nitrilotriacetic acid (Ni-NTA) resins (Qiagen). Peptidoglycan (PG) was isolated from E. coli DH10B adapted from a protocol (42) with modification. Two milliliters of overnight-grown E. coli DH10B cells (OD_600_, ∼2) was harvested and resuspended in 1 ml of 0.1 M NaCl-Tris, pH 8.0, boiled for 20 min at 100°C and washed once with 0.1 M NaCl-Tris, pH 8.0, by centrifugation at 10,000 × *g* to collect the PG pellet. Purified Tae protein (50 μg) in 100 μl 0.1 M NaCl-Tris, pH 8.0, was added to resuspend the pellet of boiled PG and incubated at 37°C for 3 h. Tae activity was heat inactivated at 100°C for 5 min, and the digested PG was harvested by centrifugation at 10,000 × *g* and resuspended in 1 ml double-distilled water (ddH_2_O) followed by trypsin and mutanolysin (Sigma-Aldrich) digestion. The muropeptides (200 μl) were reduced by 50 μl of 0.5 M sodium borohydrate for 20 mins and stopped by adding 10 μl phosphoric acid. For muropeptide detection, a linear ion trap-orbitrap mass spectrometer (Orbitrap Elite; Thermo Fisher Scientific, Bremen, Germany) coupled online with a ultrahigh-performance liquid chromatography (UHPLC) system (Acquity UPLC; Waters, Milford, MA) was used. For LC-mass spectrometry (LC-MS) analysis, solvent A with 0.1% formic acid in aqueous phase and solvent B with 0.1% formic acid in 100% acetonitrile (ACN) were used as the mobile phase for LC separation. The compounds were separated online with a reverse-phase column (BEH C18, 1.8 μm, 1.0 by 100 mm; Waters, Milford, MA) at the flow rate of 150 μl/min using gradients of 0 to 1 min, 0.5% mobile B; 1 to 9 min, 0.5 to 30% mobile B; 9 to 10 min, 30% mobile B; 10 to 10.1 min, 30 to 0.5% mobile B; and 10.1 to 11.5 min, 0.5% mobile B. The total chromatography separation time for each analysis was 11.5 min. The mass spectrometer was operated in positive ion mode and set to one full Fourier transform (FT)-MS scan (*m/z*, 50 to 2,000; resolution, 60,000).

### Growth inhibition assay of Tae.

The A. tumefaciens-E. coli coincubation conditions were identical to those mentioned in “Interbacterial competition assays” above. After coincubation, the cells were washed and resuspended in 0.9% saline at an OD_600_ of 1, which was diluted 100-fold into 3 ml LB broth with appropriate antibiotics to monitor the recovery growth of the recipient E. coli cells. The culture tubes were incubated at 37°C with shaking at 250 rpm, and the OD_600_ at different times was recorded.

### Competitiveness assays.

The coincubation conditions were identical to those mentioned in “Interbacterial competition assays” above. A. tumefaciens donor and E. coli (BW25113) recipient were mixed at a ratio of 1:9 on LB agar, and A. tumefaciens intraspecies competition was mixed as 1:1 ratio on 523 agar. After 16 h incubation, cells were washed out with 0.5 ml 0.9% saline, and the genomic DNA of the cell mixture was extracted by using the Wizard genomic extraction kit (Promega). The quantity of C58 cells in the whole bacterial population was obtained by quantitative PCR reactions with a pair of universal 16S rRNA gene primers (16S-rRNA-F/R [Table S2]) and *Agrobacterium*-specific primers for atu0231 (atu0231-F/R [Table S2]). The competitiveness of the individual C58 strain is expressed as a competitiveness index calculated as 2 − (*C_T_* of atu0231 − *C_T_* of 16S) for the strain of interest/2 − (*C_T_* of atu0231 − *C_T_* of 16S) for WT C58, where *C_T_* is threshold cycle. The index is based on the competitiveness performance in comparison to the WT C58 strain in a bacterial population. The index value close to 1 indicates similar survival performance as WT C58, <1 indicates compromised performance, and >1 suggests an advantage over WT C58. Similarly, for comparing the proportional percentage of the donor strain (*tde1* carrying) in a mixed A. tumefaciens population, universal 16S rRNA gene primers were used to reflect the total population, and a pair of *tde1*-specific primers (tde1-F/R [Table S2]) was used to measure the amount of donor strain. It was first standardized by a serial dilution of a fixed amount of C58 WT genomic DNA with the two pairs of primers, and the donor proportion was calculated as 100% × amount of *tde1* as donor cells/amount of 16S as total cells.

### Microscopy.

For cell length observation and measurement of E. coli DH10B harboring vector pTrc200, pTae, or pTaeMX, overnight cultures of these E. coli strains were each subcultured in LB medium containing spectinomycin with shaking at 37°C until an OD_600_ about 0.2 was reached. IPTG (final concentration, 0.5 mM) was added and incubated for 4 h. The cells were observed under the Zeiss Axio Imager Z1 microscope with EC Plan-Neofluar 40×/0.75 numerical aperture. Multiple images of three individual experiments were taken by using the software Zen 2.3 (Carl Zeiss Microscopy), and the cell length was measured by using ImageJ (http://imagej.nih.gov/ij/) according to the internal length standard recorded by ZEN. For cell morphology observation of E. coli coincubation with A. tumefaciens under fluorescence microscopy, recipient E. coli Δ*mepS* cells harboring plasmid pRL-GFP were grown in 523 medium to an OD_600_ of 0.5 and mixed with different A. tumefaciens strains at a 9:1 ratio. The mixtures were spotted on a 523 agar plate for 3 h at 28°C. Cells were directly taken from the plate and transferred to glass slides for observation. For Hoechst staining, the cells were first mixed with 0.1× Hoechst in phosphate-buffered saline (PBS) for 5 min prior for viewing. Fluorescence microscopy was performed on a Zeiss Axio Imager Z1 microscope equipped with an Axiochem 506 digital camera and a Plan-Apochromat 100×/0.14 Oil DIC M27 objective lens. Exposure times were typically 100 ms for differential interference contrast (DIC), 50 ms for GFP, and 400 ms for Hoechst. The experiments were performed at least in triplicate, and a representative image is shown. Images were analyzed by using ZEN and ImageJ. The cellular area of individual Δ*mepS* cells with GFP signals was automatically measured by using ImageJ and with manual confirmation.

## Supplementary Material

Supplemental file 1
