# Supplementary material for: Agrobacterium tumefaciens Deploys a Versatile Antibacterial Strategy To Increase Its Competitiveness
Source: J Bacteriol. 2021 Jan 11;203(3):e00490-20. doi: 10.1128/JB.00490-20 (PMC7811202; doi:10.1128/JB.00490-20)
Supplement: Supplemental file 1 [file supp_data_source_00490-20.pdf]

Fig S1.

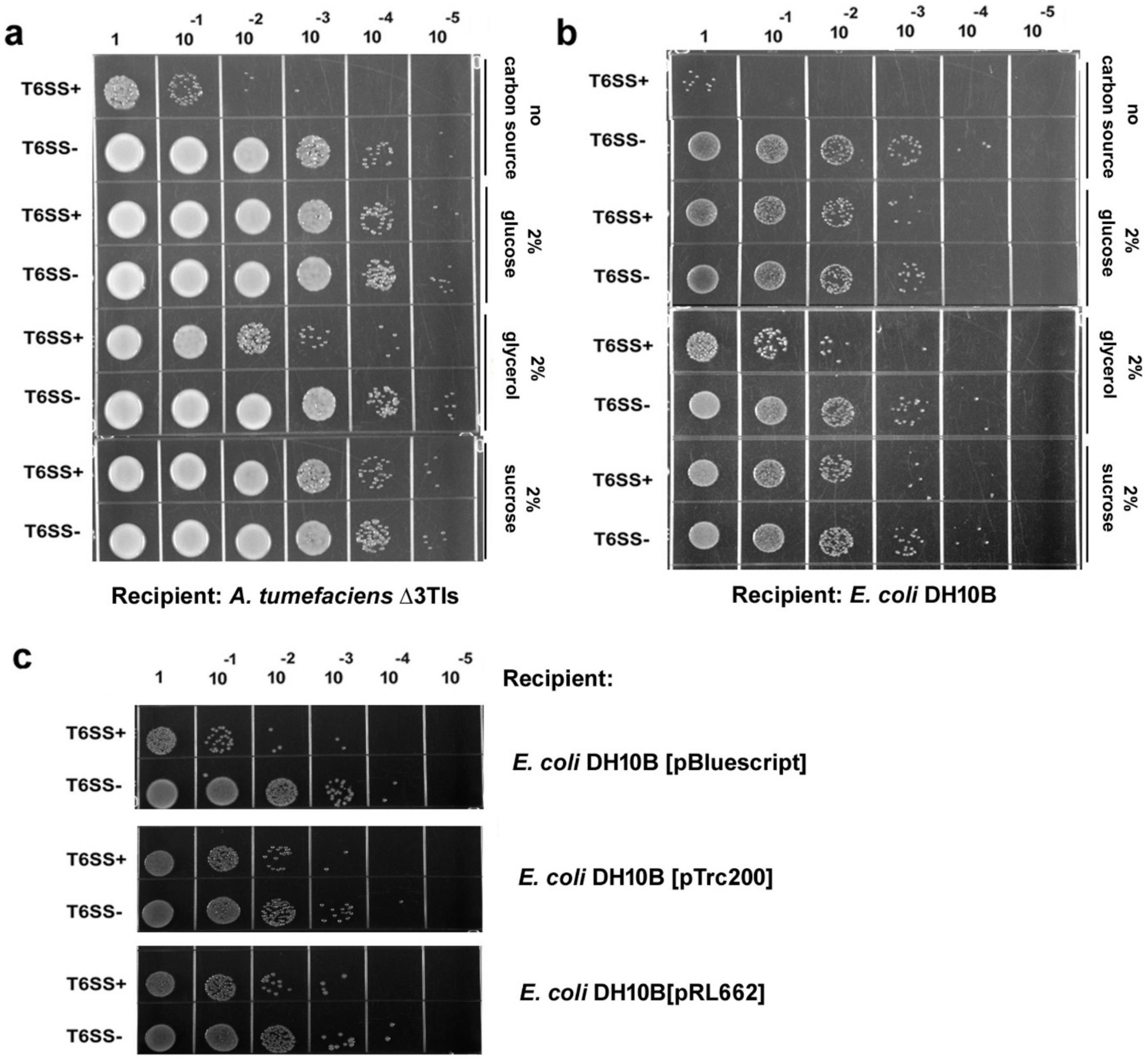

Fig S1. Variations in the co-inoculation environment affect the outcome of the T6SS dependent attack of *A. tumefaciens*. (a-b) Recovery of recipient cells (T6SS-susceptible mutant *A. tumefaciens* Δ3TIs or *E. coli*) after co-incubation with *A. tumefaciens* strain C58 with (T6SS+, i.e. WT) or without (T6SS-, i.e. *ΔtssL*) active T6SS on ABMES agar supplement with different carbon sources at ratio 30:1 (donor: recipient). The recovery of recipient cells on carbon-starved ABMES agar was significantly lower than ABMES agar supplemented with glucose and sucrose. The supplement of glycerol has less effect than glucose and sucrose in suppressing T6SS killing outcome. **(c)** Recovery of *E. coli* recipient

cells harbouring different vectors for antibiotic resistant (pBluescript for carbenicillin, pTrc200 for spectinomycin, pRL662 for gentamicin) after co-incubation with *A. tumefaciens* with (T6SS+, i.e. WT) or without (T6SS-, i.e., *ΔtssL*) active T6SS on AK medium supplemented with glucose at ratio 30:1 (donor: recipient). The recipient cells were selected on LB agar with corresponding antibiotic. Representative results of at least two independent experiments are shown.

Fig S2.

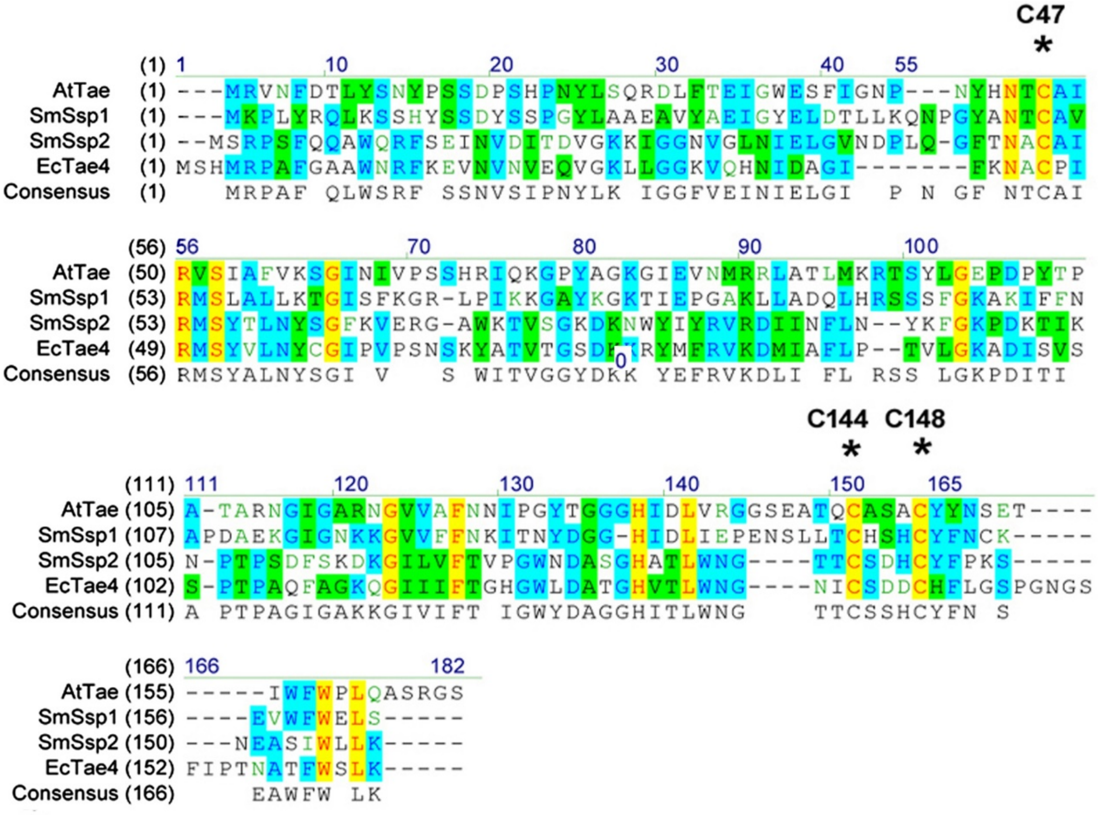

Fig S2. Amino sequence and phylogenetic analyses of AtTae and other selected Tae4 family proteins Amino sequence alignment of *A. tumefaciens* (AtTae) with *S. marcescens* SSp1 (SmSsp1), SSp2 (SmSsp2) and *Enterobacter cloacae* Tae4 (EcTae4). The identity and similarity between AtTae and SmSsp1 is 39.39% and 55.15%, respectively, but between AtTae and EcTae4 is 15.79% and 30.99%. The three conserved cysteine residues important to protein activity are highlighted (AtTae, C47, C144 and C148).

Fig S3.

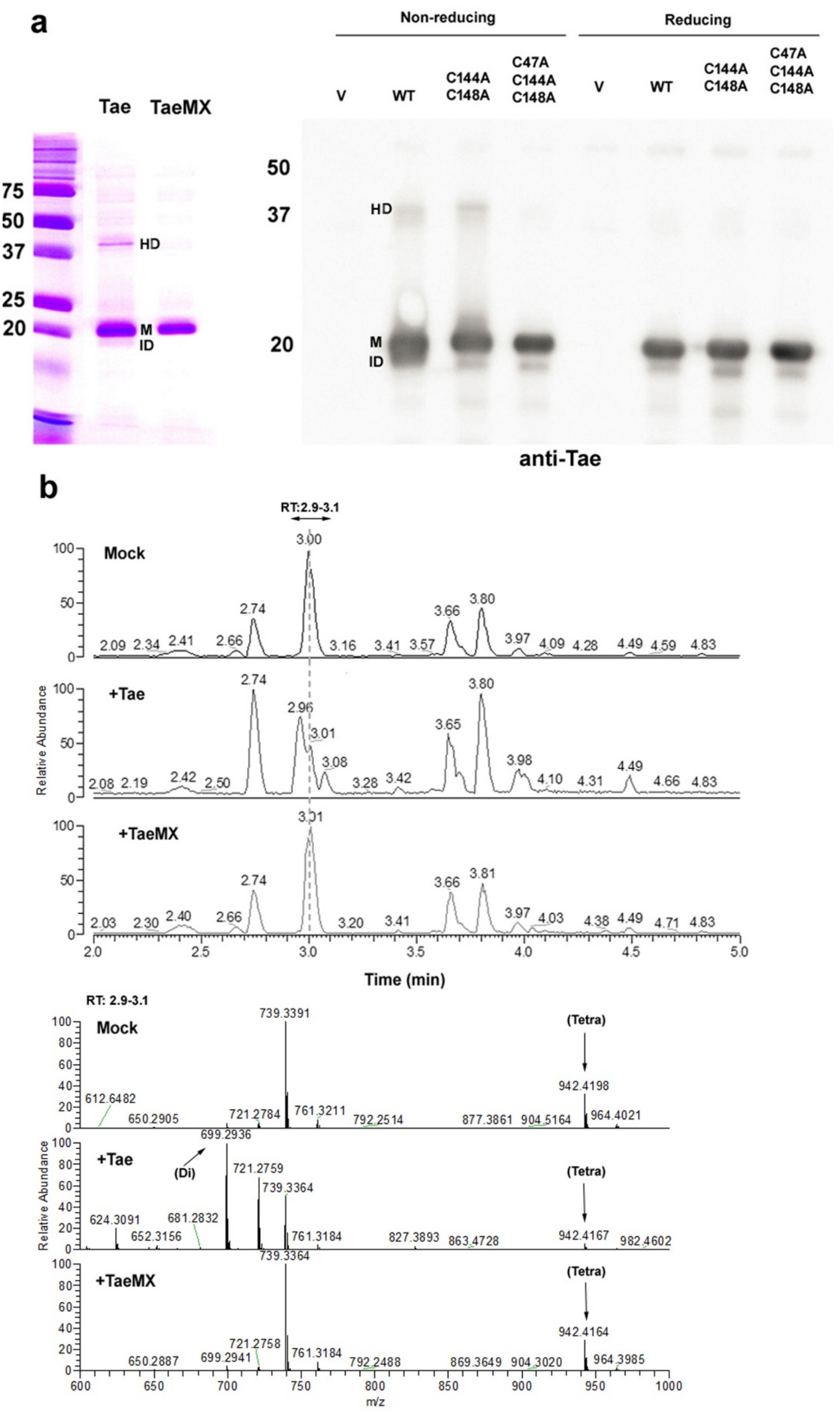
a
Tae
TaeMX
75
50
37
25
20
HD
M
ID
Non-reducing
Reducing
V
WT
C144A C148A
C47A C144A C148A
50
37
20
anti-Tae
b
RT:2.9-3.1
Mock
+Tae
+TaeMX
Relative Abundance
Time (min)
RT: 2.9-3.1
(Tetra)
(Di)
m/z

Fig S3. Protein and enzymatic analyses of purified AtTae (a) *A. tumefaciens* Tae and its loss of function of TaeMX proteins were overexpressed and purified from *E. coli* BL21(DE3) and analysed in a non-reducing SDS-PAGE. Immunoblot to detect Tae (WT) and TaeMX (C144A/C148A and C47A/C144A/C148A amino acid substitution variant) in a non-reducing and reducing SDS-PAGE. (M=monomeric form, ID=putative monomer with an internal disulphide bond, and HM=homodimer) (**b**) Base peak chromatograms and mass spectrums of AtTae-treated peptidoglycan. Peptidoglycan isolated from *E. coli* DH10B was incubated in the absence (Mock) or presence of purified Tae or TaeMX followed by mutanolysin digestion to generate muropeptides. The digested muropeptides were analysed by UPLC-MS and a specific peak (RT=2.96) was found in the AtTae-treated chromatogram and analysis in the mass spectrums indicates the enrichment of disaccharide dipeptide (Di) product (*m/z*: 699.29) and reduction of disaccharide tetrapeptide (Tetra) product (*m/z*: 942.42) in the AtTae-treated spectrum.

Fig S4.

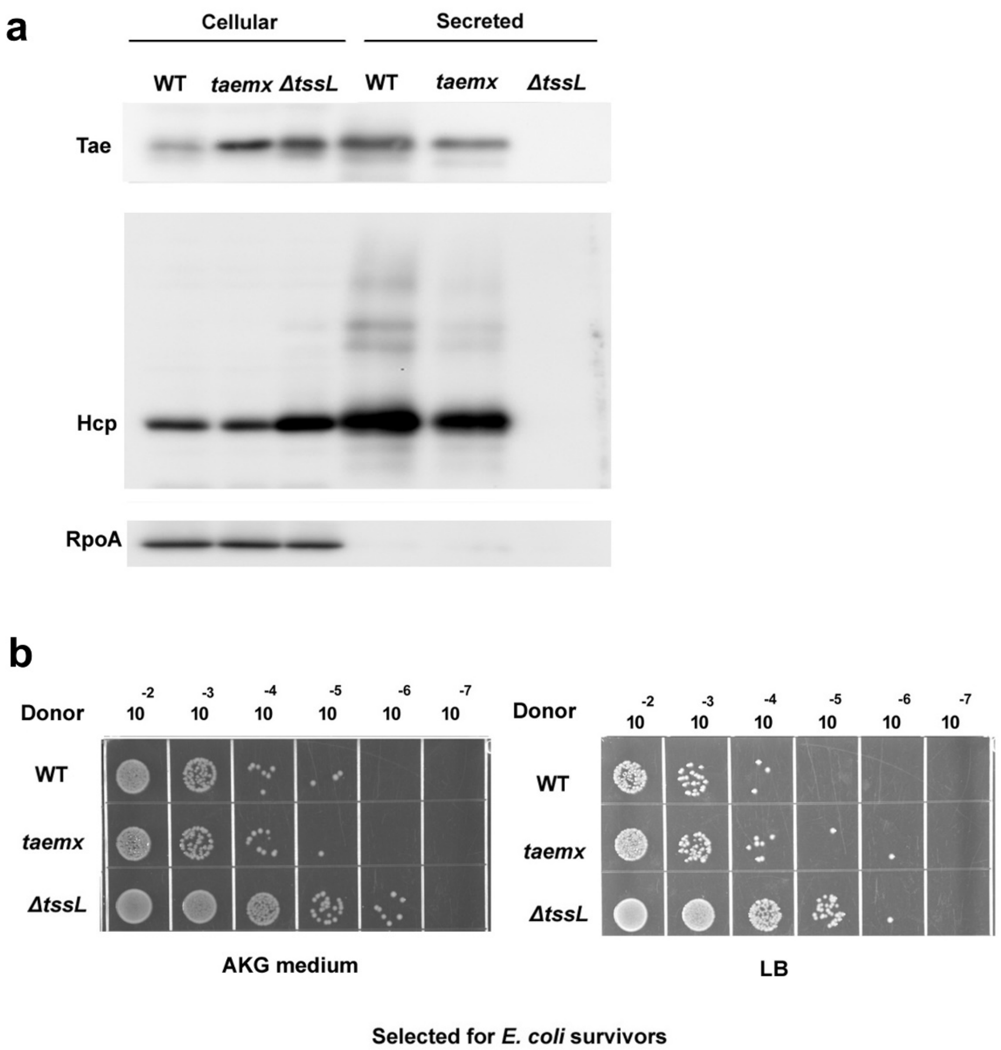

Fig S4. Secretion and antibacterial activity assays of AtTae (a) Secretion assay of *A. tumefaciens* C58 WT, *taemx*, *ΔtssL*. Immunoblots to detect cellular or secreted Tae, Hcp, and RpoA of *A. tumefaciens* strains grown in 523 medium. Hcp is positive control of T6SS secreted protein and RpoA is a loading control and negative control of secreted protein. **(b)** Recovery of *E. coli* DH10B harbouring pTrc200 after co-incubation with *A. tumefaciens* with C58 WT, *taemx*, *ΔtssL* on AKG medium or LB medium at a ratio of 10:1 (donor: recipient) for 16 hr at 28°C. The WT strain used is the WT *tae* gene recovery in Δ*tae* that underwent the same process in generating *taemx.* Representative results of at least two independent experiments are shown.

Fig S5.

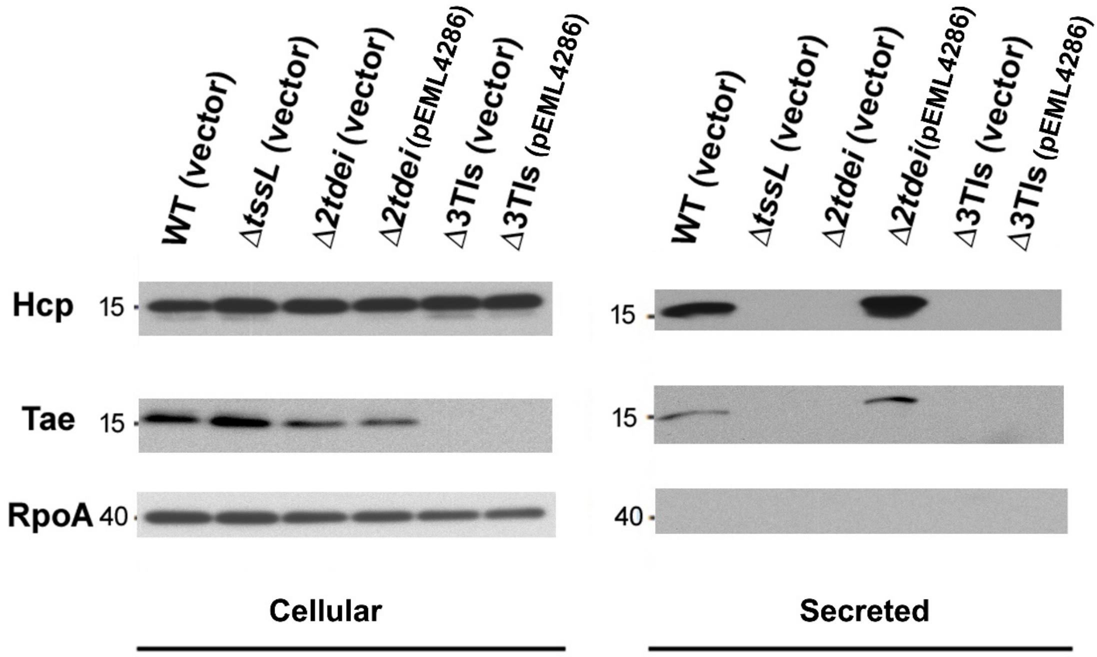

Fig S5. Secretion assays of AtTae in the absence of functional Tde effectors. Deletion of *tde-tdi1* and *tde2-tdi2* genes impairs the efficiency of Hcp and Tae secretion. Immunoblots of Hcp, Tae and RpoA of cellular or secreted fractions from various *A. tumefaciens* C58 strains grown in 523 medium. Strains with deletion of *tde-tdi1* and *tde2-tdi2* genes (*Δ2tdei*) or all three effector-immunity pairs (Δ3TIs) were complemented with a vector control (pTrc200) or pEML4286 (pTrc200 expressing *atu4349-tde1(H190A,D193A)-atu4351-atu4352*).

Fig S6.

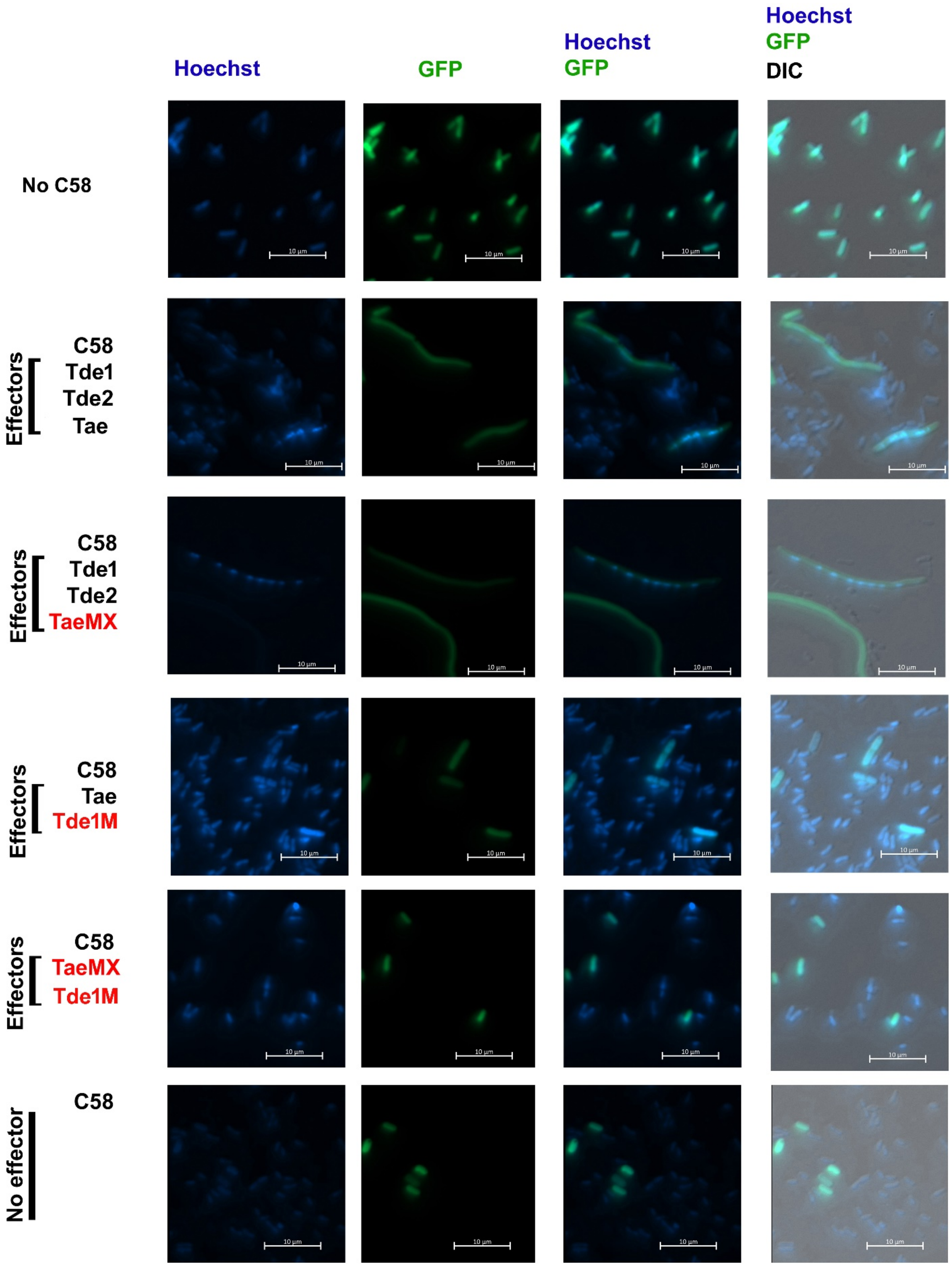

Hoechst
GFP
Hoechst
GFP
Hoechst
GFP
DIC
No C58
Effectors
C58
Tde1
Tde2
Tae
Effectors
C58
Tde1
Tde2
TaeMX
Effectors
C58
Tae
Tde1M
Effectors
C58
TaeMX
Tde1M
No effector
C58
10 µm

Fig S6. Cell morphology and DNA staining of *E. coli ΔmepS* cells after co-incubation with various *A. tumefaciens* strains deploying different effector(s). *E. coli ΔmepS* cells harbouring pRL-GFP(S65T) were co-incubated with various *A. tumefaciens* strains. *A. tumefaciens* donor cells and recipient cells (*ΔmepS*) were mixed at a 9:1 ratio and spotted on 523 agar plate for 3 hr at 28°C. After co-incubation, the cells were taken for fluorescence microscopy observation at 100x magnification. From top to bottom: *E. coli* only without donor cells (No C58); *E. coli* with WT C58 donor carrying all functional effectors (Tde1+/Tde2+/Tae+); *E. coli* with *taemx* carrying functional Tde effectors and a non-functional Tae (Tde1+/Tde2+/TaeMX+); *E. coli* with *Δ2tdei*+pEML4286 carrying a functional Tae and a non-functional Tde1 (Tae+/Tde1M+); *E. coli* with *Δ2tdei::taemx*+pEML4286 carries a non-functional Tae and a non-functional Tde1 (TaeMX+/Tde1M+) and *E. coli* with Δ3TIs lacking all three effectors (No effectors). The cells were stained with Hoechst before observation under the microscope to visualize DNA inside the cells. The text in red indicates the non-functional effector.

Fig S7.

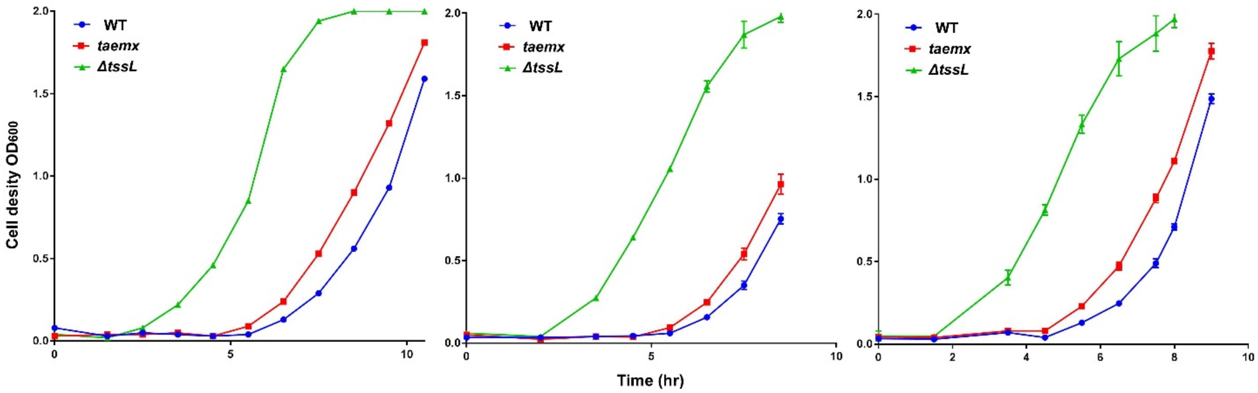

Fig S7. Growth curves of *E. coli* DH10B after co-incubation with *A. tumefaciens.* *E. coli* DH10B harbouring pTrc200 was co-cultured with *A. tumefaciens* containing a WT copy of *tae* (WT), a catalytic site amino acid substitution mutant of *tae* (*taemx*), and *ΔtssL* mutant on LB agar at ratio 10:1 (donor: recipient) for 16 hr at 28°C. A major difference in recovery time was observed between WT and *ΔtssL* mutant treated *E. coli*. As compared with WT, *taemx* treated *E. coli* demonstrated a slightly faster recovery. Curves of three independent experiments are shown.

Table S1. Bacterial strains and plasmids

Strain /plasmid	Relevant characteristics	Source/Ref.
A. tumefaciens		
C58	Wild type virulent strain containing nopaline-type Ti plasmid pTiC58	Eugene Nester
ΔtssL	*atu4333* deletion mutant, C58	(1)
Δ3tis	*atu3639-atu3640*, *atu4350-atu4351* and *atu4346-atu4347* deletion mutant, C58	(2)
Δ2tdei	*atu4350-atu4351* and *atu4346-atu4347* deletion mutant,C58	(2)
Δtae	*atu4347* deletion mutant, C58	(3)
Δtae::tae (WT)	atu4347 deletion mutant, chromosomal complemented with WT atu3437 in the same location, C58	This study
Δtae::taeMX (*taemx*)	atu4347 deletion mutant, chromosomal complemented with loss of function atu3437 (C47A, C144A and C148A) in the same location, C58	This study
Δ2tdei:taemx	*atu4350-atu4351* and *atu4346-atu4347* deletion mutant, WT *tae* is replaced by *taemx*,C58	This study
E. coli		
DH10B	F^- araDJ39 Δara, leu)7697 *ΔlacX74 galU galK rpsL deoR* ϕ80*dlacZΔ*M15 *endAI nupG recAl mcrA Δ* (*mrr hsdRMS mcrBC*)	(4)
BW25113	*rrnB Δ lacZ4787 hsdR514 Δ (araBAD)567 DE(rhaBAD)568 rph-1* (ME9062)	NBRP, Japan
ΔmepS	*ΔmepS* deletion mutant (JW2163), BW25113	NBRP, Japan
BL21(DE3)	Host for overexpressing genes driven by the T7 promoter	(5)
Δimp	Deletion of T6SS structural *imp* operon (*Δ*00817-00801), *Dd* 3937	This study
**Plasmids**		
pRL662	Vector control, GmR, broad host range vector derived from pBBR1MCS-2	(6)
pRL-GFP	GFP (S65T) inserted into pRL662	(7)
pTrc200	Vector control, SmR,SpR, pVS1 origin lacIq, *trc* promoter expression vector	(8)
pMepS	Expression vector of HA-tagged *E. coli mepS* driven by *trc* promoter in pTrc200	This study
pTae	Expression vector of HA-tgged *A. tumefaciens tae* (*atu4347*) driven by trc promoter in pTrc200	This study
pTaeMX	Expression vector of HA-tgged *A. tumefaciens tae* mutant (C47A, C144A and C148A) driven by *trc* promoter in pTrc200	This study
pTae-His	Expression vector of HA+His-tgged *A. tumefaciens tae* (*atu4347*) driven by trc promoter in pTrc200, for protein purification	This study
pTaeMX-His	Expression vector of HA+His-tgged *A. tumefaciens tae* (*atu4347*) driven by trc promoter in pTrc200, for protein purification	This study
pTai	Expression vector of HA-tagged *A. tumefaciens tai* (*atu4346*) driven by *lac* promoter in pRL662	(2)
pEML4286	Expression vector of *A. tumefaciens atu4349-4350(H190A D193A)-4351-4352* driven by *trc* promoter in pTrc200 (also known as pTrc4349-4352 (H190A D193A))	(2)

Table S2. List of primers

Primer Name	Sequence
16S-rRNA-F	5'-TGGAGCATGTGGTTTAATTCGA
16S-rRNA-R	5'-TGCGGGACTTAACCCAACA
atu0231-F	5'-CGGCGGCGGATATTCACATCAT
atu0231-R	5'-GCCTTGCCCGCATCACCGAAA
tde1-F	5'-CAGGCTGCGAATGATGTGGCA
tde1-R	5'-GAGAGCGCAATTGCTCCGATC
Not mentioned in the main text (the red text indicating the mutation site)	
tae – cloning	
NcoI-tae1-F	5'-aaaaaccatggacATGCGCGTTAACTTTGACACCCTCTA
XbaI-tae1-R	5'-aaaaatctagaGGACCCGCGGCTGGCCTGCAG
tae- mutagenesis	
Tae-C47A-F	5'-CCGAACTATCATAATACCGCCGCCATCCGCGTCAGCATC
Tae-C47A-R	5'-GATGCTGACGCGGATGGCGGCGGTATTATGATAGTTCGG
TaeC144-148A-F	5'-GGTCGGAAGCCACCCAAGCCGCGTCGGCCGCCTACTACAATTCGGAAACG
TaeC144-148A-R	5'-CGTTTCCGAATTGTAGTAGGCGGCCGACGCGGCTTGGGTGGCTTCCGACC
mepS – cloning	
NcoI-mepS-F	5'-aaaaaccATGGTCAAATCTCAACCGATTTTGAGA
XbaI-mepS-R	5'-aaaaatctagaTTAGCTGCGGCTGAGAACCCGGCG

References

1. Ma LS, Lin JS, Lai EM. 2009. An IcmF family protein, ImpLM, is an integral inner membrane protein interacting with ImpKL, and its walker a motif is required for type VI secretion system-mediated Hcp secretion in *Agrobacterium tumefaciens*. J Bacteriol 191:4316-29.
2. Ma L-S, Hachani A, Lin J-S, Filloux A, Lai E-M. 2014. *Agrobacterium tumefaciens* deploys a superfamily of type VI secretion DNase effectors as weapons for interbacterial competition in planta. Cell Host Microbe 16:94-104.
3. Lin JS, Ma LS, Lai EM. 2013. Systematic dissection of the agrobacterium type VI secretion system reveals machinery and secreted components for subcomplex formation. PLoS ONE 8:e67647.
4. Grant SG, Jessee J, Bloom FR, Hanahan D. 1990. Differential plasmid rescue from transgenic mouse DNAs into Escherichia coli methylation-restriction mutants. Proc Natl Acad Sci U S A 87:4645-9.
5. Studier FW, Rosenberg AH, Dunn JJ, Dubendorff JW. 1990. Use of T7 Rna-Polymerase to Direct Expression of Cloned Genes. Methods in Enzymology 185:60-89.
6. Vergunst AC, Schrammeijer B, den Dulk-Ras A, de Vlaam CMT, Regensburg-Tuink TJG, Hooykaas PJJ. 2000. VirB/D4-dependent protein translocation from Agrobacterium into plant cells. Science 290:979-982.
7. Heim R, Tsien RY. 1996. Engineering green fluorescent protein for improved brightness, longer wavelengths and fluorescence resonance energy transfer. Current Biology 6:178-182.
8. Schmidt-Eisenlohr H, Domke N, Baron C. 1999. TraC of IncN plasmid pKM101 associates with membranes and extracellular high-molecular-weight structures in Escherichia coli. J Bacteriol 181:5563-71.
